# Early Preclinical Studies of Ergosterol Peroxide and Biological Evaluation of Its Derivatives

**DOI:** 10.1021/acsomega.4c04350

**Published:** 2024-08-19

**Authors:** Taotao Ling, Luz V. Arroyo-Cruz, William R. Smither, Emily K. Seighman, Michelle M. Martínez-Montemayor, Fatima Rivas

**Affiliations:** † Department of Chemistry, Louisiana State University, 133 Chopping Hall, Baton Rouge, Louisiana 70803, United States; ‡ Department of Biochemistry, Universidad Central del Caribe, School of Medicine, P.O. Box 60327, Bayamón, Puerto Rico 00960-6032, United States

## Abstract

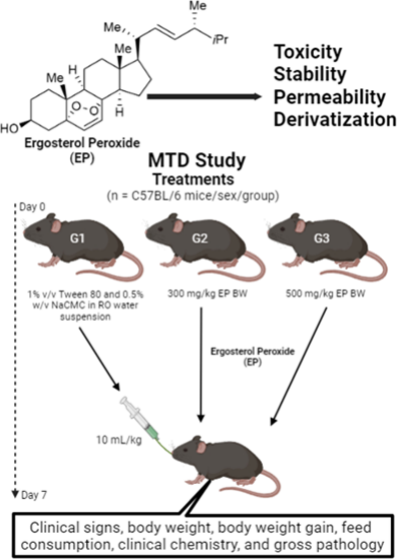

*Ganoderma lucidum* is a medicinal mushroom that produces various pharmacological compounds, including triterpenoids. A major bioactive component of *G. lucidum* is ergosterol peroxide (EP), which is attributed to its anticancer effects. The current study focuses on the *in vitro* ADME (absorption, distribution, metabolism, and elimination), *in vivo* efficacy and toxicity of EP, and the synthesis of new EP derivatives to improve aqueous solubility. It was found that EP is metabolically stable in liver microsomes and plasma. *In vivo* studies showed that EP inhibits tumor growth in murine cancer models, and it is well tolerated by mice. The maximum tolerated dose was investigated in mice at escalating doses with a defined maximum amount of 500 mg/kg, which indicated no signs of toxicity, confirmed by plasma chemistry and analysis of harvested tissues. Complementary organ toxicity assays including cardio and hepatotoxicity assays of EP demonstrated no inhibitory effects. Next, a focused library of EP derivatives was developed to investigate the iterative addition of heteroatoms to improve the aqueous solubility properties of EP. Significant solubility improvement was observed by the introduction of hydrogen bonding promoting groups, particularly the sulfate group. Superior aqueous solubility properties are directly correlated with the biological activity of the compound against triple-negative breast cancer cellular (TNBC) models. The EP derivatives maintain ample therapeutic index at the tested concentrations, indicating they engage with the same biological target(s) as the parental compound (EP). The combined studies indicate that EP and its derivatives are selective TNBC cell death inducers, while sparing noncancerous tissue.

## Introduction

Natural products remain an untapped source of molecular scaffolds for the potential development of new therapeutic agents. While natural products are often used as chemical probes for chemical biology studies, approximately 60% of clinically approved drugs have been inspired by secondary metabolites derived from natural products.^1,2^ Natural products are important tools for drug discovery due to their high molecular diversity and novel biological functionality. A wide range of these clinically approved drugs based on natural products are used for various diseases, including cancer, which continues to be a major public health problem worldwide, and it is the second leading cause of death in the United States. Moreover, breast cancer cases continue to rise accounting for 31% of female cancers in 2023.^3^ There is still a great need for effective and nontoxic therapies, specifically for patients diagnosed with aggressive subtypes, such as triple-negative breast cancer (TNBC). TNBC tumors lack the expression of therapeutic biomarkers and thus are typically treated through systemic chemotherapy (anthracyclines, taxanes, platinum-containing chemotherapeutic agents, *etc.*) in addition to surgical tumor removal.^4^ However, adverse side effects of these anticancer drugs alone or in combination are frequent, and the development of drug resistance processes leads to tumor recurrences. Therefore, the development of safer therapeutic agents is urgently needed. Accordingly, our study focuses on the studies of a promising natural product, which exhibits a broad range of biological activities and is emerging as a safe compound for healthy tissue and should offer a feasible opportunity for the development of effective agents against cancer models.

**Figure 1:**
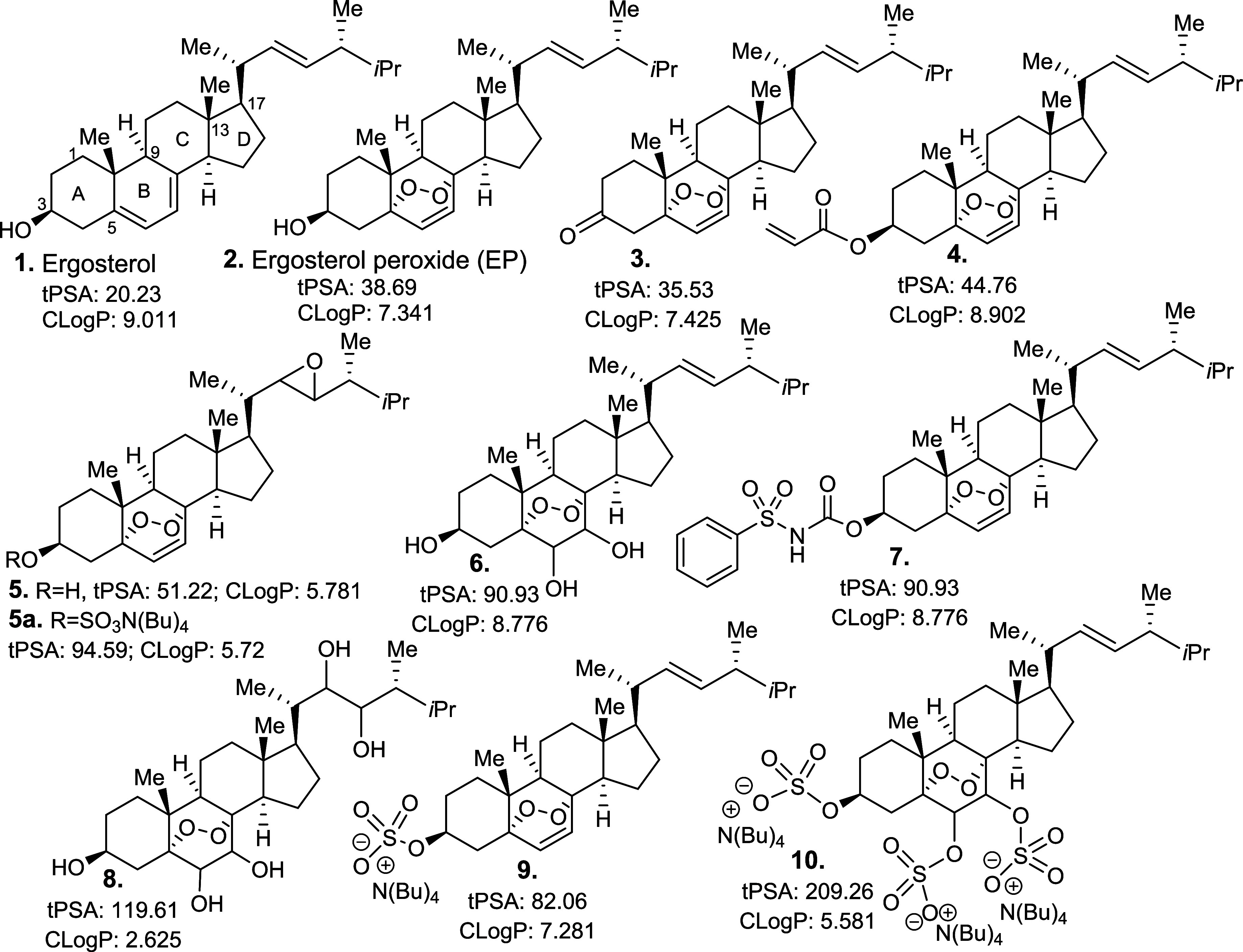
Ergosterol, ergosterol peroxide (EP), and its derivatives.

*Ganoderma lucidum* belongs to the Polyporaceae fungus family, a common species widely spread in Europe, Asia, and the American continent.^5^*G. lucidum* has a strong track record of medicinal uses throughout South Asia, mainly in Chinese medicine.^6,7^*G. lucidum* has shown a broad range of bioactive activities against human health disorders, from minor inflammatory diseases to several cancer subtypes. The bioactive constituents of *G. lucidum* range from small lipids to complex triterpenoids including ergosterol peroxide (EP, **2**, Figure 1), which is the compound focus of this study. *In vitro* EP studies demonstrate their reactive oxygen species (ROS), cytotoxicity, and apoptosis induction capacities in a variety of cancer subtype models.^8−13^

Previous studies from others and our group have demonstrated the potential of the bioactive compounds of *G. lucidum*, namely, EP. Although similar bioactive natural products used in Traditional Chinese Medicine such as triptolide show effective anticancer activities,^14^ their toxicity significantly impede their clinical application.^15^ In contrast herein, we show that EP and the generated EP derivatives demonstrate selective anticancer effects, are safe at high doses, and do not induce damage in healthy tissue. Moreover, *in vivo* efficacy of EP as a tumor reducing agent has been demonstrated in a melanoma model.^12^ However, a comprehensive assessment of the pharmacological properties (absorption, distribution, metabolism, and elimination) of EP, and overall tolerability remain unknown. These critical pharmacological parameters are required for its advancement toward preclinical animal model studies. Thus, the current investigation aims to provide the foundation of a medicinal program to advance EP toward lead optimization.

## Results

**Scheme 1:**
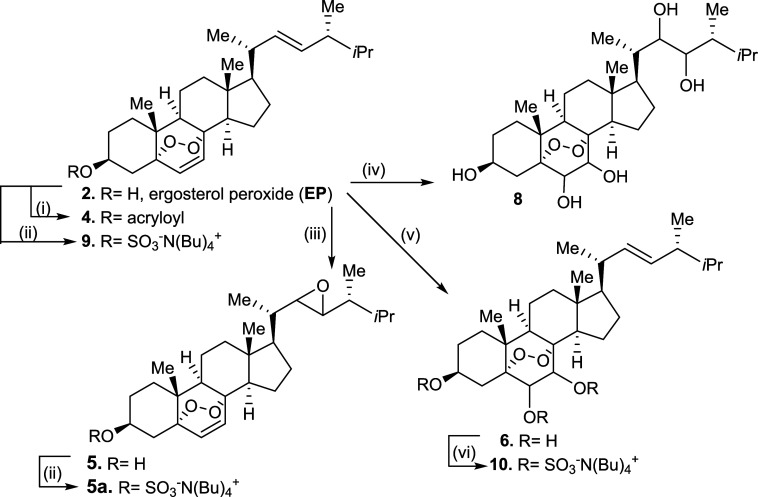
Synthesis of Compounds **4**, **5**, **6**, **8**, **9**, and **10** Reaction conditions: (i) acryloyl chloride (1.1 equiv), Et_3_N, 0 to 25 °C, 6 h; (ii) SO_3_.pyr (1.0 equiv), THF, 45 °C, 5 h; then tetrabutylammonium iodide (1.0 equiv), 45 °C, 20 min; (iii) *m*CPBA (1.1 equiv), CH_2_Cl_2_, 25 °C, 16 h; (iv) OsO_4_ (cat.), NMO (2.0 equiv), 25 °C, 12 h; (v) OsO_4_ (cat.), NMO (5.0 equiv), 25 °C, 24 h; (vi) SO_3_.pyr. (3.0 equiv), THF, 45 °C, 5 h; then tetrabutylammonium iodide (3.0 equiv), 45 °C, 20 min.

Ergosterol (**1**, Figure 1) belongs to the sterol class, and it is an essential constituent of fungi cell growth and development.^5^ Ergosterol occurs as a white or slight yellow, odorless solid, similar to its family member, EP (**2**, Figure 1). These compounds display poor aqueous solubility due to their nonpolar tetracyclic carbon system along with an alkyl side chain. EP possesses more embedded oxygen atoms, leading to improved aqueous solubility over ergosterol. To determine the therapeutic potential of EP, a focused library of compounds **3**–**10** were generated (Figure 1 and Scheme 1). Molecules that can hydrogen bond with water have higher solubility in aqueous solutions, an important factor in drug bioavailability, as it affects cellular uptake and can also directly promote the thermodynamic stability of the dispersed drug molecules. Introducing hydroxy and sulfate functional groups to enhance the hydrogen-bonding capacity of EP can improve the aqueous solubility (hydrophilicity) of the parental compound. We had previously reported the biological activity of compounds **3** and **7** against breast cancer cell models,^16,17^ and to increase the hydrophilicity scope of the EP core, an additional oxygen atom was introduced (compounds **4**–**6**, and **8**–**10**, Figure 1 and Scheme 1).

First, EP was treated with acryloyl chloride to provide compound **4** in an 85% yield. Epoxidation of EP under *m*CPBA reaction conditions led to compound **5** as an inseparable diastereomeric mixture in good chemical yields, and further sulfonation provided compound **5a** in 93% yield. Regioselective monodihydroxylation under catalytic OsO_4_ and NMO provided compound **6** after 12 h, and if the reaction was allowed to proceed for longer periods of time (24 h), it produced the bisdihydroxylated compound **8** in 87% yield. Next, sulfonation of the hydroxyl group at C-3 of EP was mediated by SO_3_.pyr treatment under heating conditions for 5 h, followed by tetrabutylammonium iodide to generate the corresponding tetrabutylammonium sulfate salt **9** in good chemical yields. The same reaction conditions were applied to generate compound **10** in an 83% yield. This focused EP library introduced several heteroatoms, enabling the identification of enough potential solubility trends. While the endoperoxide moiety is presumably responsible for the anticancer activity of EP, the global steroidal structure is likely to play a key role in target engagement.^12,17^

First, topological polar surface area (*t*PSA), a widely used molecular descriptor that provides insight into compound absorption and membrane penetration, was theoretically calculated for these compounds along with the estimated *c* log *P* (partition coefficient) or log *P* to further assess their global therapeutic potential.^18,19^ Lipophilicity, a measurable and predictable physical property, can provide practical guidance in designing compounds with improved molecular properties.^20^ The *t*PSA sums the contribution of the surface area of polar atoms, enabling us to determine whether the compounds are more likely to be cell membrane permeable. The aqueous solubility was measured by ultraviolet/visible (UV/vis) spectroscopy.^21^ Ergosterol is a highly hydrophobic molecule as it does not contain sufficient heteroatoms and therefore has a high cLogP and low *t*PSA. EP shares an improved cLogP, but still relatively high from a desirable drug candidate (2.5–3.5). However, the overall physicochemical properties of compounds **1** and **2** are similar due to their overall shape, hydrophobicity, hydrogen bonding, and charge distribution, leading to low aqueous solubility. EP and derivative **3** showed similar properties, but ketone **3** is slightly more potent than EP. Compound **4** has a higher *c* log *P*, while compounds **5**–**10** have lower *c* log *P* values. To determine LiPE (lipophilicity efficiency), the following formula was used: LiPE = *p*IC_50_ – log *P*, according to established methods.^22,23^ First, the EC_50_ of these compounds using the cell models SUM149, MDA-MB-231, and BJ was determined *via* the 72 h cytotoxicity CellTiter-Glo assay (CTG assay), Table 1. The obtained EC_50_ values for the newly generated EP derivatives were within a similar range for other derivatives previously described against TNBC models.^17^ In addition, *p*IC_50_ values were calculated as follows: *p*IC_50_ = *X* – log_10_(EC_50_), where *X* is a function of molarity (*X* = 6, when EC_50_ is in the micromolar range).

**Table 1:** Properties of Compounds **1**–**10**^a^

num	SUM149 (EC_50_)	MDA-MB231 (EC_50_)	BJ	TI (BJ/SUM149)	*p*IC_50_	log *P*	LIPE	solubility limit (μg/mL)
**1**	61	>120	ND	ND	4.21	6.93	–2.72	0.59
**2**	6	18	>50	>9	5.22	6.51	–1.28	21.42
**3**	5	7	>50	>9	5.3	6.84	–1.54	10.65
**4**	30.98	47.57	92.53	>2.5	4.5	7.42	–2.91	24.12
**5**	2.74	15.69	70.41	>25	5.56	5.42	0.14	5.56
**5a**	2.24	7.37	48	>15	5.64	5.72	–0.07	26.2
**6**	14.53	25.45	134.8	>9	4.83	4.92	–0.08	23.12
**7**	5.6	12	47.1	>8	5.25	7.86	–2.6	26.76
**8**	6.42	29.65	171.6	>26	5.19	3.19	2	24.82
**9**	2.70	3.47	50.02	>18	5.56	6.81	–1.24	15.54
**10**	3.11	10.68	77.85	>24	5.51	5.58	–0.07	17.83

a (EC_50_) values are in micromolar.

**Figure 2:**
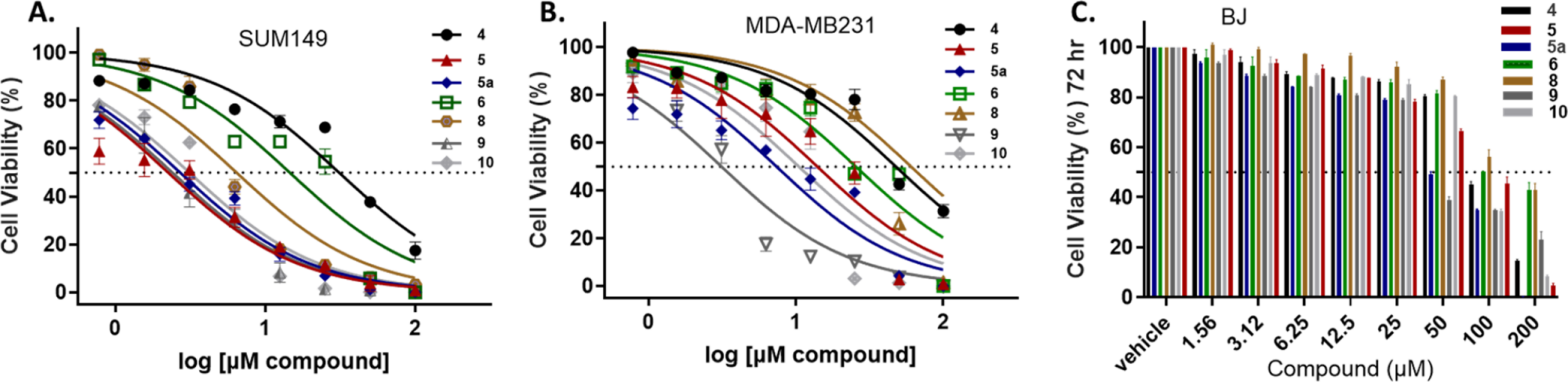
Cytotoxicity viability assay (CTG assay, 72 h of drug exposure) of compounds **4**–**10** against various cell line models was performed to assess cytotoxicity. (A) SUM149, (B) MDA-MB-231, (C) BJ. Data are expressed as mean ± SD from three independent experiments.

The newly generated EP compounds showed an improved cytotoxicity profile against TNBC cell models with a therapeutic index (TI > 2) when compared to the parental EP compound (Figure 2). The data set indicates that these compounds have improved properties against the SUM149 cell model (Figure 2A), with similar properties to EP for MDA-MB-231 cells (Figure 2B). Importantly, these compounds maintain selectivity toward cancerous cell models while sparing the noncancerous BJ cell model at the tested concentrations (Figure 2C). Compounds **5**–**5a**, **9**, and **10** demonstrated the most therapeutic potential (Table 1).

The high carbon-to-heteroatom ratio in steroidal compounds renders them less likely to demonstrate good aqueous solubility, which can affect the compounds’ bioavailability. However, the introduction of hydroxy and sulfate salt groups, particularly sulfate salt groups, can improve the physicochemical properties of the compounds. These compounds had an increase in hydrophilic interactions as their heteroatom content was augmented. Notably, the sulfate group increases the water solubility, which would allow them to be more readily transported into the cell. There are various natural steroidal compounds such as estrogen and androgens, which are sulfonated during their preparation as a protecting step and are hydrolyzed on need basis by steroid sulphatase.^24^ Thus, these sulfate functional groups can serve as prodrug mechanisms to enhance solubility and be released by this enzyme in a biological setting.

LiPE was determined (Table 1) for these compounds with the calculated *p*IC_50_ from the SUM149 assay, and compound **8** provided the optimal outcome with a number of 2. Empirical evidence indicates that high-performing oral drug candidates have a high LiPE (>6).^22^ Our study found that the newly generated compounds **5**–**6** and **8**–**10** were better than EP, while modest improvement was observed from the aqueous solubility assay as the solubility for these compounds remained within the 20 μg/mL range as EP (Table 1).

Next, the stability of EP was evaluated (Table 2). The prodrug EP derivative **7** was included to compare with EP. *In vitro* metabolic stability and plasma binding affinity studies provide an estimate of *in vivo* compound stability and potential hepatic clearance along with reported controls^25−29^ (Table 2). The clearance of EP was evaluated through microsomal assay in mouse and human species (see the Supporting Information (SI) for details). Remarkably, EP displayed a robust clearance with a half-life of ―*t*1/2 = 5.63 and 2.74 h in mouse and human hepatic microsomes, respectively. Conversely, compound **3** showed rapid clearance with a half-life of ―*t*1/2 = 0.90 h and *t*1/2 = 0.83 h, respectively, in both murine and human models. Next, plasma protein binding of EP was studied because the free drug is responsible for both efficacy and toxicity. Optimizing plasma protein binding as an independent parameter does not significantly influence efficacy, and it must be considered as one unit in a multifactorial system. Interestingly, EP showed optimal plasma binding affinity toward mouse and human plasma proteins 86 and 72%, respectively, while compound **3** showed a much higher affinity in both species, which is not a favorable parameter.^22^ However, both compounds **2** and **3** displayed good plasma stability (>48 h).

**Table 2:** *In Vitro* Stability and Plasma Binding Assay for EP

	metabolic stability (mouse)			metabolic stability (human)			plasma binding (mouse)		plasma binding (human)		plasma stability (mouse)		plasma stability (human)	
control or compound	*t*1/2 (h)	STD	clint (mL/Min/K g)	*t*1/2 (h)	STD	clint (mL/Min/K g)	protein binding (%)	STD	protein binding (%)	STD	t1/2(h)	STD	t1/2 (h)	STD
verapamil	1.11	0.09	51.42	1.36	0.1	15.31	92.67	0.56	92.32	0.43				
eucatropine											1.87	0.08	0.41	0
2											>48		>48	
7	0.9	0.06	63.82	0.83	0.06	24.91	99.83	0.08	99.38	0.39	>48		>48	

To further study the properties of EP, stability and permeability are shown in Table 3. The simulated gastric fluid (SGF) assay is an important element in determining the overall drug-like potential of compounds.^29,30^ Anecdotal studies of oral consumption of extracts by humans indicate positive outcomes of digestion of mixtures of EP and its related natural products found in *G. lucidum*.^2^ The SGF assay is designed to measure the susceptibility of pepsin under acidic conditions to metabolize the compound, and results typically mimic the observed vulnerabilities of a compound *in vivo* as demonstrated by the known control.^31^ Chlorambucil was used as positive control as previously described.^32,33^ EP had a half-life (*t*_1/2_) greater than 48 h, while compound **3** displayed a *t*_1/2_ of close to 9 h. Next, EP was subjected to the Parallel Artificial Membrane Permeation Assay (PAMPA), and control,^34,35^ low permeability was observed at 65.31 × 10^–6^ cm/s, while attempts to measure compound **3** were unsuccessful as the drug partitioned onto the plate and membrane surfaces—thereby rendering accurate log Pe calculations for compound **3** not feasible. Caco-2 permeability studies were conducted to validate and to provide information on EP’s ability to cross the intestinal barrier and its potential for interactions with drug transporters.^36−39^ The rate of transport in both directions (A-B and B-A) across the cell monolayer enables an efflux ratio (B-A/A-B) to be determined, enabling us to determine if a compound undergoes active efflux (efflux ratio ≥2). EP’s A-B and B-A permeability were promising, as shown in Table 3, similar to the control compound carbamazepine with an efflux ratio of close to 1. However, compound **3** showed poor absorption, and therefore a lower bioavailability of this compound would be expected.

**Table 3:** *In Vitro* EP Stability and Permeability Assays

	SGF stability		PAMPA					Caco-2 permeability				
control or compound	*t*1/2 (h)	STD	pH	Avg Pe (10–6 cm/s)	SD Pe	%*R*	SD R	AVG Papp A/B (nm/s)	SD Papp A/B	AVG Papp B/A (nm/s)	SD Papp B/A	efflux ratio (B2A/A2B)
chlorambucil	18.43	0.35										
verapamil			7.40	2267.00	0.9	26.0	22.80					
carbamazepine								375.73	34.01	277.27	14.89	0.74
2	>48		7.40	65.31		81.90	25.95	342.21	247.74	319.20	138.94	0.93
3	8.92	0.57	7.40	00	00			6.26	5.20	3.07	1.76	0.49

Two *in vitro* experiments were performed to assess EP’s toxicity, and these were focused on two important organs, the heart and the liver. Cardiotoxicity hERG studies were conducted to test EP’s potential toxic effects on potassium channels that are essential for normal electrical activity in the heart.^40^ Our data shows that under the conditions tested, EP did not reach an IC_50_ when tested up to 30 μM compared to the pharmacological potassium hERG channel blocker, E-4031 (Table 4).

**Table 4:** hERG Channel Inhibition Assay of EP

compound	% mean inhibition						IC_50_ determination			
vehicle	first addition	second addition	3rd addition	fourth addition	fifth addition	sixth addition	IC_50_ (μM)	*p*IC_50_	*p*IC_50_ SE	*N*
	3.55	3.63	3.73	5.5	7.81	6.67				3
EP	0.1 μM	0.3 μM	1 μM	3 μM	10 μM	30 μM	IC_50_ (μM)	*p*IC_50_	*p*IC_50_ SE	*N*
	1.99	2.99	3.04	6.21	10.13	18.72	>30	<4.52	0.01	5
E-4031	0.001 μM	0.003 μM	0.011 μM	0.033 μM	0.1 μM	0.3 μM	IC_50_ (μM)	*p*IC_50_	*p*IC_50_ SE	*N*
	–0.79	4.64	22.09	56.44	87.31	96.79	0.03	7.57	0.06	3

To test for potential hepatotoxicity effects, cytochrome P450 enzyme inhibition assays were performed. Hepatoxicity studies demonstrate that EP would need to circulate in plasma at a level higher than 9.9, 16.3, or 19.9 μM, to have inhibitory action on CYP2C8, CYP1A2, or CYP2C19 enzymes, respectively, or it would require to reach these concentrations to initiate potential problems with any comedications that are metabolized by these specific Cytochrome P450 enzymes (Table 5) and the reported controls.^41−45^

**Table 5:** Cytochrome P450 Inhibition Assay^a^

inhibitor	test concentration	CYP3A4-Midazolam	CYP3A4-Testos-terone	CYP2C9	CYP2D6	CYP1A2	CYP2C8	CYP2B6	CYP-2C19
EP	0.025–25 μM	>25	>25	>25	>25	16.3	9.9	>25	19.9
ketoconazole	0.01–10 μM	0.023	0.015						
sulfaphenazole	0.01–10 μM			0.184					
quinidine	0.01–10 μM				0.074				
α-naphthoflavone	0.001–1 μM					0.003			
quercetin	0.05–50 μM						0.658		
ticlopidine	0.05–50 μM							0.335	1.158

a The data represent the average of three independent replicas.

Next, we conducted experiments to assess EP tolerability *in vivo*. MTD results show that the oral administration of EP at doses of 300 and 500 mg/kg revealed no abnormal clinical signs in mice. Body weight (Tables S1 and S2) and percent body weight gain (data not shown) of EP-treated mice were statistically comparable to the control group. Average feed intake (Tables S3 and S4) of EP-treated mice was comparable to the control group. Clinical chemistry analytes (see Table S5 for parameters) of EP-treated animals were statistically comparable to control group animals except a minor, but statistically significant, decrease of 6% in albumin at 300 mg/kg EP (G2), and a decrease in triglycerides by 19% at 500 mg/kg EP (G3) was observed only in male mice when compared with vehicle control G1. However, these changes observed at 300 and 500 mg/kg were not considered as toxicologically significant,^40,46^ and the changes were not dose-related. The values obtained were within the normal range for the species (Table S6A,B). Importantly, all treated animals survived the study termination without any negative physical observable effect. Gross pathological observations of all of the mice did not reveal any abnormality upon external or internal examination (Table S7A,B).

**Figure 3:**
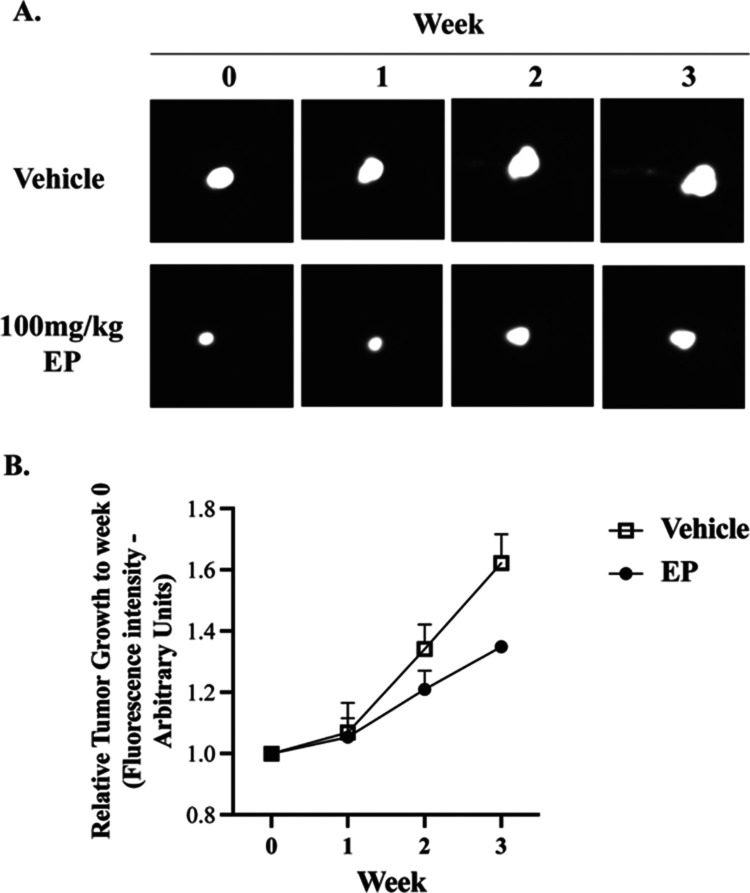
Short-term *in vivo* EP effects in a TNBC model. MDA-MB-231-GFP cells (5.0 × 10^5^) were injected into the lower right mammary fat pad of female hairless severe combined immunocompromised mice (SHO-SCID). (A) Fluorescent intravital image analysis of primary tumors started from injection day. Treatment [vehicle or ergosterol peroxide (EP) ip administered 3 times per week] started on week 1. The image shows tumor progression of one representative mouse tumor out of 3 per treatment group. (B) Mammary tumor growth was quantified as changes in the integrated density of GFP fluorescence relative to week 0. Results show that EP significantly decreases (*P* < 0.05) tumor growth. Mean ± SEM of three representative mice per treatment.

Finally, to test the effect of EP on breast cancer progression *in vivo*, we established mammary fat pad tumors from GFP-tagged TNBC MDA-MB-231 cancer cells as we have previously described.^47^ Mice were injected with TNBC cells diluted in Matrigel in their fourth mammary fat pad, and when tumors were palpable, the mice were injected *via* intraperitoneal (i.p.) 3 times per week with vehicle (10% ethanol) or 100 mg/kg BW of EP. This concentration is below the MTD that we report herein and showed efficacy in a different tumor model,^12^ highlighting the therapeutic potential of EP. The progression of the tumor was quantified by fluorescence image analysis obtained on a weekly basis. The values provided include the relative tumor area, which was calculated as the fluorescence intensity of each tumor on week of imaging relative to the fluorescence intensity of the same tumor on week 0 as previously described.^48^Figure 3 indicates that there is a significant time *x* treatment interaction (*P* < 0.05), where EP decreases tumor growth by week 3.

## Discussion

This study demonstrates that an increase in heteroatoms to the EP steroidal core, particularly sulfur/oxygen atoms, positively improves its solubility properties, which leads to superior EP derivative compounds. The initial assessment of the pharmacological properties (ADME) of EP is therapeutically promising and establishes benchmarks against cancer models. Further improvements in ADME properties during lead optimization^40,49^ will be sought, while preserving the potency and selectivity of EP. The findings indicate that EP does not reach the necessary levels in plasma to cause cardiotoxicity and hepatotoxicity, which are two important parameters to consider during the evaluation of new drug candidates. For example, a blockage of the hERG potassium ion channels by the evaluated drugs could result in the induction of lethal cardiac arrhythmias.^41^ Furthermore, in the case of the hepatic cytochrome P450 enzymes, these are a polymorphic multigene enzyme family that play a role in the metabolism of drugs, steroids, and other compounds.^42^

Our results show EP has favorable nontoxic effects, rendering it suitable for further preclinical development. Our earlier target-based drug discovery studies demonstrate that EP targets the Ubiquitin Protein Ligase E3 Component N-Recognin 4 (UBR4) in two TNBC cell lines.^17^ Future research efforts are focused on elucidating EP interactions with the target by measuring their associated biological reactions. Herein, we demonstrate that EP as a single agent had no abnormal effects on body weight, feed consumption, clinical chemistry, or gross pathology in either male or female mice treated with up to 500 mg/kg of EP. Moreover, we disclose the *in vivo* effects of EP. In a short-term efficacy study, EP-treated mice showed reduced tumor growth compared to the vehicle-treated mice by week 3 of treatment. At the end of the study, EP-treated mice presented with a statistically significant tumor size reduction (∼17%), indicating the therapeutic value of EP. Furthermore, there was no onset of early death or observable negative effects due to treatment. Hence, it can be concluded that for EP administration as a single oral dose to male or female mice, the fact that the maximum tolerated dose exceeds 500 mg/kg provides confidence that long-term studies at lower dosages should be well tolerated in animal models.

## Conclusions

Natural products have served as indispensable molecular scaffolds for the discovery and development of current chemotherapeutics.^50^ Our preliminary results on EP indicate that this natural product has good potential to be further developed into a preclinical candidate.

This study found that EP is a good hit compound with favorable therapeutic properties. The combined data indicates EP and its derivatives are selective cancer cell (TNBC models) death inducers, while sparing noncancerous tissue. EP reduces tumor growth at 100 mg/kg BW, and it is well tolerated at 500 mg/kg well beyond its expected therapeutic dosage and does not display acute *in vivo* toxicity in murine models. Cardiotoxicity is a major side effect of anticancer treatments, so hERG studies were conducted. No negative effects were recorded at the tested EP concentrations. Furthermore, no significant changes in liver function were observed as determined by ALT/AST assays, and clearance due to P450s inhibition is expected to be a minor factor as partial inhibition was observed only at higher doses. To overcome EP’s poor aqueous solubility, we generated a focused EP library including S and O atoms such as compounds **5a** and **9**, which displayed superior biological activity, attributed to their improved aqueous solubility properties that facilitate cellular uptake. Further EP optimizing will involve the introduction of other heteroatoms to increase their aqueous solubility while maintaining their selectivity toward cancer cell models.

## Materials and Methods

### General Experimental Chemistry procedures

Detailed information on the general experimental chemistry procedures and ^1^H and ^13^C NMR spectra are provided in the Supporting Information.

### Cell-Based General Cytotoxicity Evaluation

The following stable human cell lines were utilized for this study: SUM149 and MDA-MB-231 (breast cancer models) and BJ (human normal fibroblast, foreskin). Cells were purchased from BioIVT or the American Type Culture Collection (ATCC). Cells were cultured in F-12-Nutrient Mixture (SUM149), DMEM (MDA-MB-231), or EMEM (BJ) medium supplemented with 10% fetal bovine serum (Sigma-Aldrich, St. Louis, MO) and 2 mmol of l-glutamine, at 37 °C in a humidified 5% CO_2_ atmosphere (no antibiotics) as indicated by the manufacturer. Cell cultures were grown to 80–90% confluence before use, except where otherwise specified. Our cell lines are authenticated every 3 months using the CellCheck service (Idexx BioResearch, Westbrook, ME). Cells were tested for mycoplasma prior to use (MycoAlert detection Kit Lonza LT07–318) and discarded if they tested positive. A cell proliferation assay was performed using the CellTiter-Glo (Promega Corp., Madison, WI) luminescent cell viability assay kit. Cells were detached with trypsin, counted, and seeded in white polystyrene flat-bottom sterile 96-well tissue culture-treated plates (catalog no. 3917, Corning, Glendale, Arizona) and incubated overnight at 37 °C. MDA-MB231, SUM149, and BJ cells were seeded into 96-well plates at concentrations experimentally determined (0.4 × 10^4^, 0.25 × 10^4^, and 0.3 × 10^4^ cells per well, respectively) to ensure logarithmic growth during the duration of the experiment and to prevent adverse effects on cell growth by DMSO exposure. The plates were incubated for 12 h before treatment. Stock solutions of test compounds (10 mM in DMSO) in nine 3-fold serial dilutions were dispensed. The final concentration of DMSO was 0.3% (v/v) in each well. The positive control used was staurosporine (10 μM) as the kill-all control, and the negative control used is DMSO as we have published. Staurosporine is a potent alkaloid inducer of various cell death modalities but primarily apoptosis, making it a reliable positive control for viability assays. The plates were incubated for 72 h and then quenched with CTG at 50 μL per well at RT. Plates were then incubated at RT for 20 min and centrifuged at 1000 rpm for 1 min. Luminescence was read on a CLARIOstar Plus plate reader (BMG LabTech, Ortenberg, Germany).

### General Solubility Procedure

Briefly, clear 96-well PS plates with lids (catalog no. 3903, Corning, Glendale, Arizona) were used for spectrophotometric determination of absorbance with a CLARIOstar Plus plate reader (BMG LabTech, Ortenberg, Germany) to determine at which concentration the compounds are precipitating out of solution. Decrease in transmittance of 2% or higher was considered significant. Compound solutions of 10 mM in DMSO were prepared followed by a serial dilution series, which were vortexed to ensure the solutions were homogeneous. The 96-well plate was then prepared with distilled H_2_O and allowed to equilibrate for 1.5 h. Then, the plate was placed in a CLARIOstar Plus plate reader or Cytation C10 (Biotek, Agilent) and set to detect absorbance at 220–700 nm. Each well was read individually. Three replicate assays were conducted for each experimental condition, and a minimum of three independent experiments were conducted.

### Plasma Stability Assay

Briefly, pooled blood from 3 animals for experiment (male Balb/c 6–8w from the division of laboratory animal medicine in-house breeding colony) was collected, and the heparinized plasma was prepared. Plasma and test compounds were added to individual wells of a 96-well microtiter plate. Compounds were incubated at 37 °C for the provided time points. All tests were performed in triplicates. The test compound was incubated with plasma at six different time points. The reaction was terminated by methanol containing an internal standard. After centrifugation, the concentration of the test compound in the supernatant was quantified by LC-MS/MS. Additional protocol information on compound testing is available in the Supporting Information.

### Metabolic Stability Assay

This assay provides important information on the metabolic liability of early drug discovery compounds on the basis of human and/or mouse microsomes.^51,52^ Human and/or mouse microsomal degradation was determined using multiple time points to monitor the rate of disappearance of the parent compound during incubation following the method described by Di et al.^51,52^ We used pooled human liver microsomes, mixed gender (female and male), 1.0 mL at 20 mg/mL protein (XenoTech LLC, catalog #H0620) or pooled female mouse liver microsomes (CD-1), 0.5 mL at 20 mg/mL protein (Gentest Animal Pooled Liver Microsomes, catalog #452702). The metabolic stability was evaluated *via* the half-life from the least-squares fit of the multiple time points based on first-order kinetics. Waters ACQUITY-TOQ UPLC-MS-MS-UV system was used to analyze the samples as established in various MS core facilities.^51,52^

### Parallel Artificial Membrane Permeability Assay (PAMPA)

This assay is to analyze the permeability of various drugs/compounds on a homogeneous artificial lipid membrane using the normal Double-Sink PAMPA protocol. Then, 6 μL of 10 mM compound solution in DMSO was applied to each well in a stock plate. Compounds were diluted 200-fold in PBS buffer (pH = 7.4). 180 μL of diluted solution was added to a donor plate (pION INC, Woburn, MA). A filter plate (acceptor plate; pION INC, Woburn, MA) containing 200 μL of acceptor sink buffer (ASB, pH = 7.4; pION INC, Woburn, MA) was then placed over the donor plate. The plates were incubated at room temperature for 0.5 h with magnetic stirring in individual wells to allow the compounds to cross the membrane. Fractions were collected from both the donor plate and the acceptor plate, and the concentrations were assessed by UV spectrometry (230–500 nm). Sample preparation, sample analysis, and data processing are fully automated using a Biomek FX ADME-TOX workstation and the UV-based PAMPA Evolution-96 Command Software. All compounds were tested in triplicates.

### Plasma Protein Binding Assay

This assay determines the plasma protein binding of a drug or compound and free drug concentration in plasma using rapid equilibrium dialysis. We use the Single-Use RED (rapid equilibrium dialysis, Thermo Scientific) devices to allow the aqueous component of plasma containing the free drug/compound through a dialysis membrane (MWCO ∼ 8000). Sample quantification is determined using a Waters ACQUITY-SOQ UPLC-MS-ELDS-UV system.

### CaCo-2 Permeability Assay

It was performed in the 96-well Transwell system with a modified method.^36^ Caco-2 cells were maintained at 37 °C in a humidified incubator with an atmosphere of 5% CO_2_. The cells were cultured in MEM containing 20% FBS in 75 cm flasks, 100 units/mL penicillin, and 100 μg/mL of streptomycin. The Caco-2 cells were seeded onto inserts of a 96-well plate at a density of 0.5 × 10^5^ cells/insert and cultured in the MEM containing 20% FBS for 7 days. Each cultured monolayer on the 96-well plate was washed twice with HBSS/HEPES (10 mM, pH 7.4). The permeability assay was initiated by the addition of each compound solution (10 μmol/L) into inserts (apical side, A) or receivers (basolateral side, B). The Caco-2 cell monolayers were incubated for 2 h at 37 °C. Fractions were collected from receivers (if apical to basal permeability) or inserts (if basal to apical permeability), and concentrations were assessed by UPLC/MS (Waters; Milford, MA). All compounds were tested in triplicates. The A → B (or B → A) apparent permeability coefficients (Pappa, cm/s) of each compound were calculated using the equation Pappa = d*Q*/d*t* × 1/*AC*_0_. The flux of a drug across the monolayer is d*Q*/d*t* (μmol/s). The initial drug concentration on the apical side is *C*_0_ (μmol/L). The surface area of the monolayer is *A* (cm^2^).

### UPLC/MS System

LC-MS chromasolv-grade acetonitrile (ACN) was purchased from Fisher Scientific (Pittsburgh, PA). LC-MS chromasolv-grade and formic acid were obtained from Sigma-Aldrich (St. Louis, MO). Milli-Q water, an ultrapure laboratory-grade water, was used in the aqueous mobile phase. Chromatographic separation was performed on an Acquity UPLC BEH C18 1.7 m, 2.1 × 50 mm^2^ column (Waters Corp., Milford, MA) using an Acquity ultraperformance liquid chromatography system. Data were acquired using Masslynx v. 4.1 and analyzed using the Quanlynx software suite. This was coupled to an SQ mass spectrometer. The total flow rate was 1.0 mL/min. The UPLC column was maintained at 65 °C. Solvent A was 0.1% formic acid in Milli-Q H_2_O, and solvent B was 0.1% formic acid in ACN. Samples were eluted from the column under a gradient condition. The mass spectrometer was operated in positive-ion mode with electrospray ionization. The conditions were as follows: capillary voltage 3.4 kV, cone voltage 30 V, source temperature 150 °C, desolvation temperature 350 °C, desolvation gas 750 L/h, cone gas 25 L/h. A full scan range from *m*/*z* 110 to 1000 in 0.2 s was used to acquire MS data. A single ion recording mass spectrometer for each compound was used to determine the quantification of the samples.

### Toxicity Studies

To assess potential cardiotoxicity, we performed the human ether-go-go-related gene (hERG) inhibition assay. EP effects were tested on the hERG cardiac ion channel, expressed in mammalian cells (HEK-293) using the electrophysiology platform QPatch HTX. Briefly, EP was tested at six concentrations (cumulative concentration–response). The percent change in hERG tail-current was calculated and used, where appropriate, to calculate an IC_50_ value (test compound concentration that produces 50% inhibition). For hepatotoxicity determination, we performed the microsomal CYP inhibition assay. Briefly, EP was incubated at seven increasing concentrations with pooled human liver microsomes. A probe substrate concentration, including tacrine (CYP1A2), bupropion (CYP2B6), amodiaquine (CYP2C8), tolbutamide (CYP2C9), mephenytoin (CYP2C19), dextromethorphan (CYP2D6), midazolam, and testosterone (CYP3A4), was optimized, and EP was tested with selective CYP inhibitors as positive controls and DMSO (0.3%) as the negative control. The supernatants were analyzed by LC-MS/MS.

### Maximum Tolerated Dose (MTD)

The study was conducted according to published and acceptable experimental conditions reported.^53^ Animal protocols approved by LSU#IACUCAM21065 and UCC# 037–2021–16–01-PHA-IBC. Briefly, male and female Balb/c mice were given a single oral dose administration of ergosterol peroxide to C57BL/6 female and male mice (5–8 weeks old), followed by 7 days postdose observations. Toxicity is defined as a 20% loss of body weight. The study comprised three groups for a toxicity phase including: one control and two EP-treated groups (G2 and G3), having 6 mice/sex/group. Mice from groups G2 (300 mg/kg BW) and G3 (500 mg/kg BW) were administered an EP uniform suspension (light yellow), once by oral gavage. The mice from the control group (G1) received a 1% v/v Tween 80 and 0.5% w/v NaCMC in the RO water suspension. The dosing volume was kept constant at 10 mL/kg for each mouse. Then, animals were euthanized, and organs (liver, lung, heart, and kidney) were collected to check global signs of toxicity. Parameters evaluated included observation for clinical signs, body weight, body weight gain, feed consumption, clinical chemistry, and gross pathology. The total animals per MTD was 36 mice.

### *In Vivo* Efficacy Study

Female (3-week-old) severe combined immunodeficient (SCID) Hairless Outbred (SHO) mice (Charles River Laboratories, Wilmington, MA) were segregated into two groups (*n* = 3 mice/group) and housed under specific pathogen-free conditions. The mice received an autoclaved AIN 76-A phytoestrogen-free diet (Tek Global, Harlan Teklad, Madison, WI) and water *ad libitum*. Cell inoculations were performed under isoflurane inhalation as we previously described^47^ to produce orthotopic tumors as described in the provided reference.^54^ Briefly, MDA-MB-231–GFP (5 × 10^5^ cells) were injected into the lower right mammary fat pad in a 100 μL volume of a 1:1 dilution of reduced growth factor Matrigel (BD Biosciences, San Jose, CA) and serum-free DMEM media. GFP-tagged cells were monitored weekly for the duration of the study using an iBox Explorer^2^ (UVP, CA) animal imaging system. The mice were randomly sorted according to their weight before pharmacological treatment. At 1-week post-tumor injection, the mice were injected i.p. 3 times per week with vehicle (10% ethanol) or 100 mg/kg BW of EP in 100 μL for 3 weeks. Images were acquired with an Olympus DP71 digital camera (Olympus, Waltham, Massachusetts), as described by us.^54^ Tumor fluorescence intensities were analyzed using ImageJ software (National Institutes of Health, Bethesda, MD). All animal experiments were reviewed by the Institutional Animal Care and Use Committee (assurance #D16–00343) at the Universidad Central del Caribe (UCC) at Bayamón, PR.

### Statistical Analysis

Three or four technical replicates were conducted for each experimental condition, and a minimum of three independent experiments were conducted for each assay. *In vitro* ADME values were expressed as mean ± standard deviation (SD) of at least three independent experiments. One-way analysis of variance (ANOVA) was adopted for comparisons among groups using the GraphPad Prism 10.2.3 (GraphPad Software, San Diego, CA). A difference of *P* < 0.05 was considered as statistically significant. Data are expressed as mean ± SEM. For CTG, the mean luminescence of each experimental treatment group was normalized as a percentage of the mean intensity of untreated controls (DMSO as vehicle). EC_50_ values were calculated from dose–response curve fitting *via* nonlinear regression using GraphPad Prism 10.2.3 (GraphPad Software, San Diego, CA). A therapeutic index (TI) between normal and tumor cell lines can be determined (EC_50_ non-neoplastic cell line BJ)/(EC_50_ cancer cell line). *In vivo* studies were analyzed using mixed-effects regression models to compare the changes in tumor volume over time by treatment group. Tumor volume reduction was compared using ANOVA with repeated measures, and all data was considered significant when *P* < 0.05.

## Supporting Information Available

Experimental procedures; animal information; and NMR data (PDF)

## Abbreviations

EP: ergosterol peroxide
TNBC: triple-negative breast cancer
hERG: human ether-a-go-go-related gene
ADME: absorption, distribution, metabolism, and elimination
MTD: maximum tolerated dose
CTG: CellTiter-Glo
