# Supplementary material for: Early Preclinical Studies of Ergosterol Peroxide and Biological Evaluation of Its Derivatives
Source: ACS Omega. 2024 Aug 19;9(35):37117–27. doi: 10.1021/acsomega.4c04350 (PMC11375702; doi:10.1021/acsomega.4c04350)
Supplement: Supplementary file 1 — ao4c04350_si_001.pdf [file ao4c04350_si_001.pdf]

Early preclinical studies of ergosterol peroxide and biological evaluation of its derivatives

Taotao Ling[a], Luz V. Arroyo-Cruz[b], William R. Smither[a], Emily K Seighman[a], Michelle M. Martínez-Montemayor[b*], and Fatima Rivas[a*]

[a] *Department of Chemistry, Louisiana State University, 133 Chopping Hall, Baton Rouge, LA 70803, USA;* [b]*Department of Biochemistry, Universidad Central del Caribe, School of Medicine, P.O. Box 60327, Bayamón, P.R. 00960-6032.*

RECEIVED DATE (automatically inserted by publisher); email: frivas@lsu.edu or michelle.martinez@uccaribe.edu

Supporting Information Available

I. Biological assays and supplemental tables 1-7
II. Experimental chemistry procedures 8-11
III. ^{1}H and ^{13}C NMR Spectra 12-18

I. Biological assays

Plasma stability assay: Briefly, pooled blood from 3 animals for experiment (male Balb/c 6-8w from the division of laboratory animal medicine in-house breeding colony) was collected, and the heparinized plasma was prepared. Plasma and test compounds were added to the individual wells of a 96-well microtiter plate. Compounds were incubated at 37˚C for the provided time points. All tests were performed in triplicates. Then, the test compound was incubated with plasma at six different time points. The reaction was terminated by the addition of methanol containing an internal standard. After centrifugation, the concentration of the test compound in the supernatant was quantified by LC-MS/MS. The percentage of test compound remaining at each time point relative to the 0-minute sample is reported as shown in **Graph S1**. Control compounds are known to be metabolized by plasma esterases,[1] and were also incubated with each batch of test compound.

A standard protocol and MS method was used for the plasma stability experiment. The extraction procedure for plasma/tissue samples and the spiked plasma/tissue calibration standards were identical: A 25 µL of study sample or spiked plasma/tissue calibration standard was added to individual pre-labeled micro-centrifuge tubes followed by 100 µL of internal standard prepared

[1] Racané L, Cindrić M, Zlatar I, Kezele T, Milić A, Brajša K, Hranjec M. Preclinical in vitro screening of newly synthesised amidino substituted benzimidazoles and benzothiazoles. J Enzyme Inhib Med Chem. 2021 Dec;36(1):163-174. doi: 10.1080/14756366.2020.1850711. PMID: 33404264; PMCID: PMC7801115.

in Acetonitrile (Albendazole, 500 ng/mL) was added except for blank, where 100 µL of Acetonitrile was added. Samples were vortexed for 5 minutes. Samples were centrifuged for 5 minutes at a speed of 4000 rpm at 4°C. Following centrifugation, 100 µL of clear supernatant was transferred in 96 well plates and analyzed using LC-MS/MS mode. The LC system comprises of a binary pump that can mix two mobile phases: A = Water+0.1% formic acid; B = acetonitrile +0.1%formic acid. The system was operated in gradient mode using a 6 minute program that changed mobile phases from 10-90% A to B in 0-1.2 min, 60-40% A to B in 1.2-5 min, 10-90% A to B in 5.5-6 min, and then hold at this gradient, using a Phenomenex Luna, 150 X 4.6 mm, 5 µm column. Source Parameter: positive, Ion Spray Voltage 5500, Temperature 550C, interface heater on.

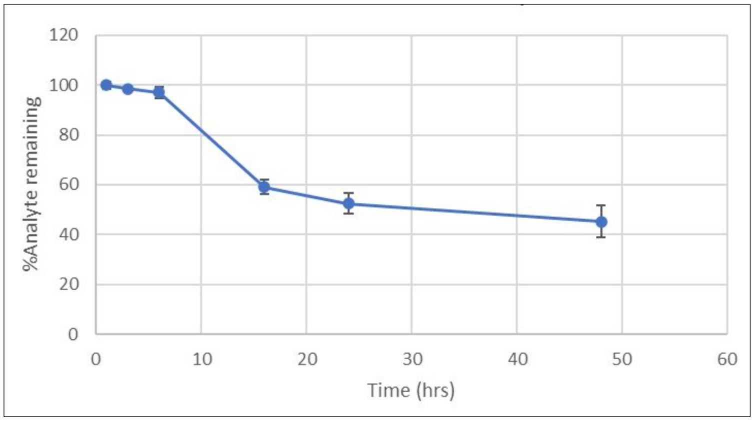

Graph S1. EP stability in mice plasma.

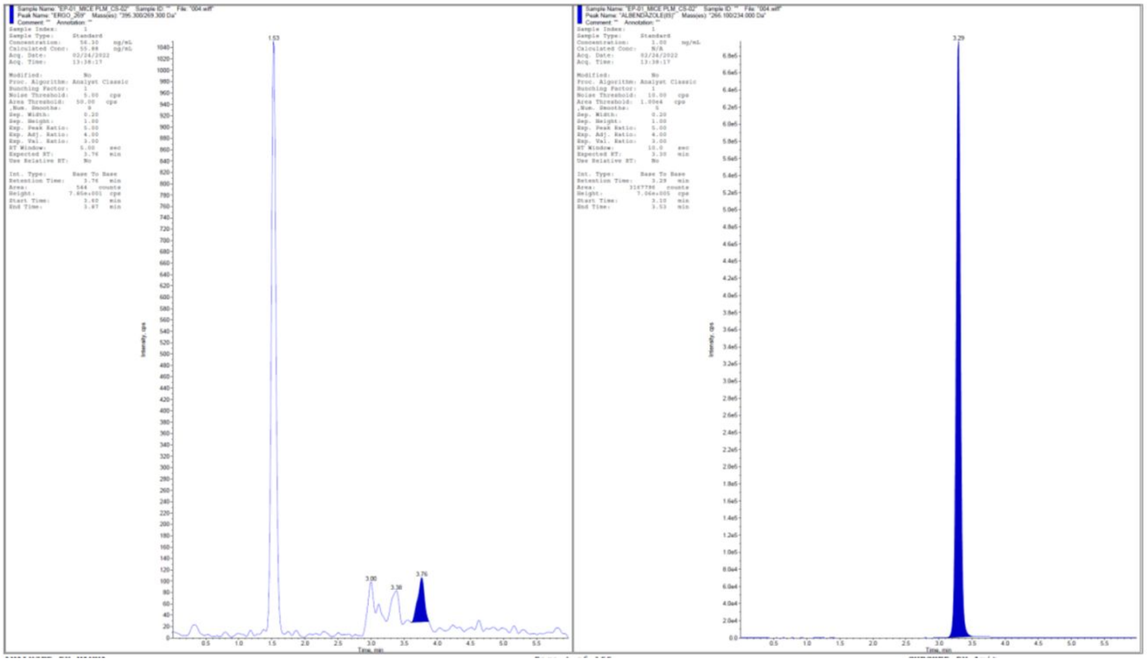

Graph S2. Representative chromatograms of the Lower Limit of Quantification of EP, which was 56.30 ng/mL (left) and albendazole as the internal standard. Retention Time (in min) for EP= 3.76 Internal Standard Albendazole= 3.29. The internal standard was used at 1 ng/mL.

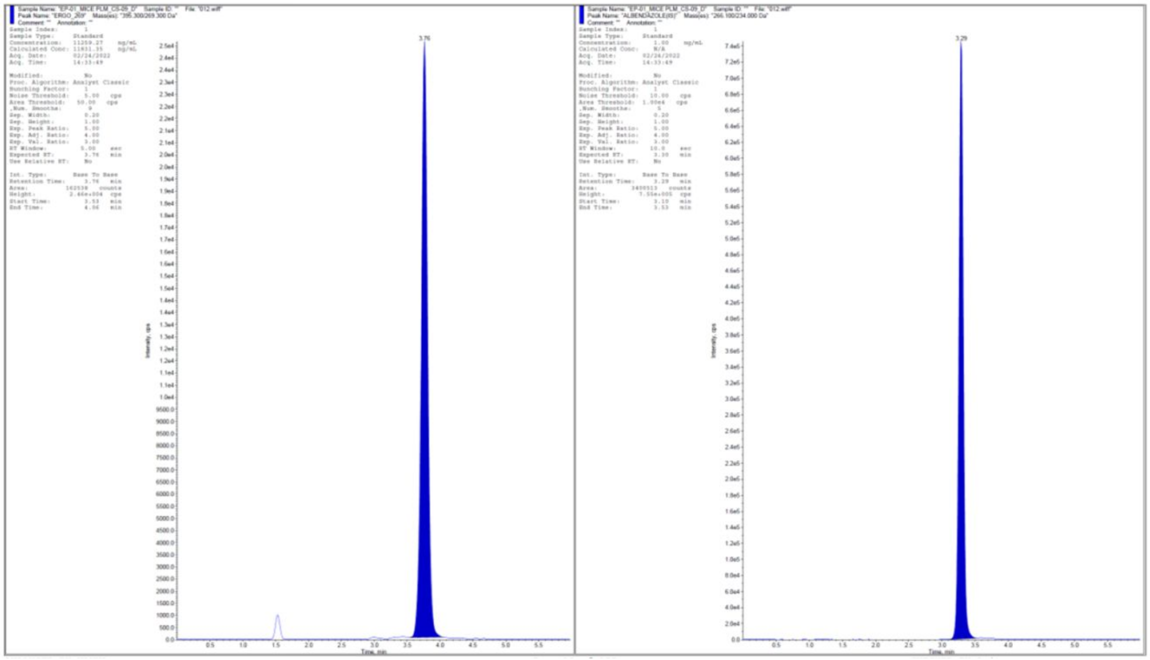

Graph S3. Representative chromatograms of the Upper Limit of Quantification of EP, which was 11259.27 ng/mL (right). Retention Time (in min) for EP= 3.76 Internal Standard Albendazole= 3.29. The internal standard was used at 1 ng/mL.

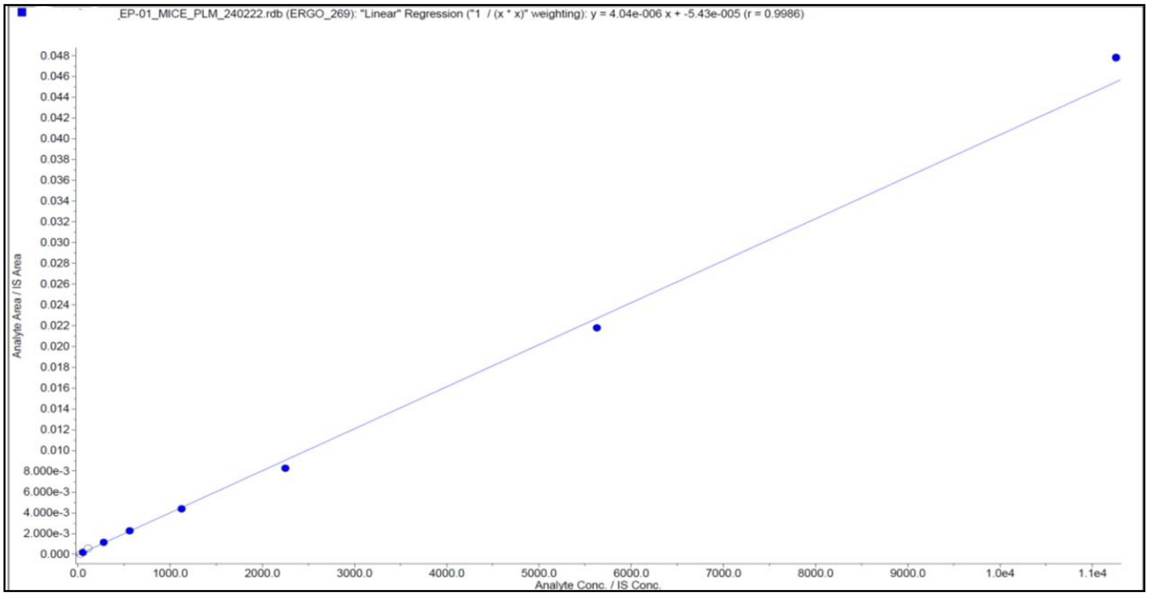

Graph S4. Representative calibration curve of EP in mice plasma.

Supplemental Tables:

Table S1. Summary - Body Weight (g) – Male.

**Mean/SD/N**		**Treatment Day**		
		**1**	**4**	**7**
**Group: G1**	**Dose: 0 mg/kg**			
**Mean**		22.63	22.33	22.72
**SD**		1.19	1.16	0.92
**N**		6	6	6
**Group: G2**	**Dose: 300 mg/kg**			
**Mean**		21.85	22.28	23.03
**SD**		1.64	1.49	1.40
**N**		6	6	6
**Group: G3**	**Dose: 500 mg/kg**			
**Mean**		22.62	22.70	22.27
**SD**		1.01	1.00	0.70
**N**		6	6	6

Key: N = Number of animals.

Table S2. Summary - Body Weight (g) – Female.

**Mean/SD/N**		**Treatment Day**		
		**1**	**4**	**7**
**Group: G1**	**Dose: 0 mg/kg**			
**Mean**		20.42	20.95	20.87
**SD**		1.59	1.82	1.40
**N**		6	6	6
**Group: G2**	**Dose: 300 mg/kg**			
**Mean**		21.05	21.15	21.45

**SD**	2.01	1.93	1.86
**N**	6	6	6
**Group: G3 Dose: 500 mg/kg**			
**Mean**	21.27	21.17	21.53
**SD**	1.50	1.71	1.42
**N**	6	6	6

Key: N = Number of animals.

Table S3. Summary - Food Consumption (g/Animal) – Male.

**Average Feed Intake/ Animal/ N**	**Treatment Day**	
	**1 to 4**	**4 to 7**
**Group: G1 Dose: 0 mg/kg**		
**Average Feed Intake/ Animal**	12.6	13.1
**N**	2	2
**Group: G2 Dose: 300 mg/kg**		
**Average Feed Intake/ Animal**	14.9	14.6
**N**	2	2
**Group: G3 Dose: 500 mg/kg**		
**Average Feed Intake/ Animal**	10.4	13.7
**N**	2	2

Key: N = Number of Cages.

Table S4. Summary - Food Consumption (g/Animal) – Female.

**Average Feed Intake/ Animal/ N**	**Treatment Day**	
	**1 to 4**	**4 to 7**
**Group: G1 Dose: 0 mg/kg**		
**Average Feed Intake/ Animal**	12.9	11.5
**N**	2	2
**Group: G2 Dose: 300 mg/kg**		
**Average Feed Intake/ Animal**	12.3	12.2
**N**	2	2

Group: G3	Dose: 500 mg/kg		
Average Feed Intake/ Animal		12.3	11.8
N		2	2

Key: N = Number of Cages.

Table S5. Parameters for clinical chemistry.

No.	Parameter	Unit
1	Alanine aminotransferase (ALT)	U/L
2	Albumin (ALB)	g/dL
3	Albumin Globulin Ratio (A:G) (calculated)	-
4	Alkaline phosphatase (ALP)	U/L
5	Aspartate aminotransferase (AST)	U/L
6	Blood urea nitrogen (BUN)	mg/dL
7	Creatinine (CREA)	mg/dL
8	Globulin (GLOB) (calculated)	g/dL
9	Glucose (GLU)	mg/dL
10	Low Density Lipoprotein Cholesterol (LDLC)	mg/dL
11	Total Bilirubin (TBIL)	mg/dL
12	Total Protein (TP)	g/dL
13	Triglycerides (TGL)	mg/dL
14	Urea (calculated)	mg/dL

Table S6A. Clinical chemistry values for male mice.

	ALT	ALB	ALP	AST	BUN	CREA	TGL
	U/L	g/dL	U/L	U/L	mg/dL	mg/dL	mg/dL
**G1 0 mg/Kg**							
**Mean**	59.82	3.09	399.99	83.55	17.49	0.20	58.22
**SD**	27.52	0.12	55.90	27.85	1.81	0.03	3.15
**N**	6	6	6	6	6	6	6
**G2 300mg/Kg**							
**Mean**	34.83	2.91*	312.51	47.80	16.45	0.17	51.23
**SD**	11.22	0.09	64.79	23.81	2.09	0.03	0.09
**N**	6	6	6	6	6	6	6
**G3 500mg/Kg**							
**Mean**	54.07	3.03	412.86	91.50	17.06	0.17	46.91*
**SD**	24.31	0.16	123.51	35.77	3.67	0.04	0.29
**N**	6	6	6	6	6	6	6

Key: G= group, N = Number of animals, *Mean value of group significantly decreased from control group at $p<0.05$.

Table S6B. Clinical chemistry values for female mice.

	ALT	ALB	ALP	AST	BUN	CREA	TGL
	**U/L**	**g/dL**	**U/L**	**U/L**	**mg/dL**	**mg/dL**	**mg/dL**
**G1 0 mg/Kg**							
**Mean**	41.51	3.02	418.31	76.90	19.53	0.21	38.93
**SD**	23.81	0.08	71.32	29.67	2.18	0.01	12.26
**N**	6	6	6	6	6	6	6
**G2 300 mg/kg**							
**Mean**	44.99	3.08	394.32	88.20	16.45	0.21	38.15
**SD**	14.53	0.21	90.58	14.90	3.15	0.02	3.96
**N**	6	6	6	6	6	6	6
**G3 500 mg/Kg**							
**Mean**	47.50	3.03	400.51	75.14	17.63	0.20	30.51
**SD**	14.47	0.11	48.77	15.22	3.05	0.02	4.79
**N**	6	6	6	6	6	6	6

Key: G=group, N = Number of animals.

Table S7A. Summary – Gross Pathology Findings – Male.

Group	G1	G2	G3
Dose (mg/kg)	0	300	500
Number of Mice Examined	6	6	6
Mode of Death			
Terminal Sacrificed	6	6	6
External Abnormalities			
No Abnormality Detected	6	6	6
Internal Abnormalities			
No Abnormality Detected	6	6	6

Table S7B. Summary - Gross Pathology Findings – Female.

Group	G1	G2	G3
Dose (mg/kg)	0	300	500
Number of Mice Examined	6	6	6
Mode of Death			
Terminal Sacrificed	6	6	6
External Abnormalities			
No Abnormality Detected	6	6	6

Internal Abnormalities			
No Abnormality Detected	6	6	6

II. Experimental chemistry procedures

General procedures

All reactions were carried out in an argon atmosphere with dry solvents, under anhydrous conditions, unless otherwise noted. Dry tetrahydrofuran (THF), toluene, diethyl ether (Et_2O), ethyl acetate (EtOAc) and methylene chloride (CH_2Cl_2), acetonitrile (CH_3CN), dimethylsulfoxide (DMSO), *N*, *N* dimethylformamide (DMF) and isopropanol (*i*-PrOH) were purchased in anhydrous form and used without further purification. Yields refer to chromatographically and spectroscopically (^{1}H NMR) homogeneous materials, unless otherwise stated. Reagents of the highest available quality were purchased and used without further purification unless otherwise stated. Reactions were monitored by thin-layer chromatography (TLC) on 0.25 mm E. Merck silica gel plates (60F-254) using UV light for visualization and an ethanolic solution of anisaldehyde and heat as developing agents. Reactions were also monitored by using Agilent 1100 series LCMS and a low-resonance electrospray model (ESI) with UV detection at 254 nm.

Title compounds were purified by flash column chromatography using E. Merck silica gel (60, particle size 0.040–0.063 mm) or Biotage Isolera Four with normal-phase silica gel. ^{1}H and ^{13}C NMR spectra were recorded on a Bruker (AV-400 or DRX-500 MHz) NMR spectrometer instruments calibrated with residual undeuterated solvent ($CDCl_3$: ^{1}H=7.26 ppm, ^{13}C=77.16 ppm; acetone-d_6: ^{1}H=2.05 ppm, ^{13}C=29.84 ppm; CD_3CN: ^{1}H=1.94 ppm, ^{13}C=1.32 ppm; CD_3OD: ^{1}H=3.31 ppm, ^{13}C=49.00 ppm; DMSO-d_6: ^{1}H=2.50 ppm, ^{13}C=39.5 ppm) as an internal reference. The following abbreviations were used to designate the multiplicities: s=singlet, d=doublet, t=triplet, q=quartet, quin=quintet, hept=heptet, m=multiplet, br=broad. Infrared (IR) spectra were recorded on a Perkin-Elmer100 FT-IR spectrometer. High-resolution mass spectra (HRMS) were recorded on an Agilent ESI-TOF (time of flight) mass spectrometer using MALDI (matrix-assisted laser desorption ionization) or ESI (electrospray ionization) or a Waters Xevo G2 Q-ToF mass spectrometer. Compounds were analyzed by using electrospray ionization in positive-ion mode. Purity of final compounds was > 95% based on analytical HPLC and NMR analysis. Yields refer to chromatographically and spectroscopically (^{1}HNMR) homogeneous materials.

Procedures and Compound Characterizations

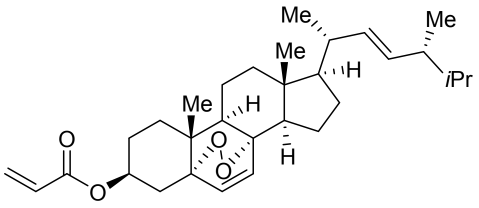

Compound 4: To a solution of ergosterol peroxide (**EP**, 42.0 mg, 0.10 mmol) in dry CH_2Cl_2 (1.0 mL) was added triethylamine (30 mL, 0.2 mmol), followed by acryloyl

chloride (9.0 µL, 0.11 mmol) at 0°C, the reaction mixture was then warmed up to 25°C and stirred at this temperature for 6 h, the reaction progress was monitored by TLC until completion. The reaction mixture was then diluted with brine (1.0 mL) and extracted with EtOAc (3 x 1.0 mL). The combined organic phase was dried over $MgSO_4$ and concentrated under reduced pressure. The crude product was subjected to silica gel column chromatography (Hexane:EtOAc, 9:1 to 7:3, v/v) to provide compound **4** (41.0mg, 0.085mol, 85% yield) as clear oil. HRMS (ESI-TOF) calculated for $C_{31}H_{47}O_4$ ($[M + H]^+$): 483.3474, found: 483.3477. ^1H NMR (400 MHz, $CDCl_3$) δ 6.51 (d, *J* = 8.5 Hz, 1H), 6.42 – 6.39 (m, 1H), 6.36 (s, 1H), 6.23 (d, *J* = 8.5 Hz, 1H), 6.08 (dd, *J* = 17.4, 10.5 Hz, 1H), 5.82 – 5.78 (m, 1H), 5.25 – 5.04 (m, 4H), 2.20 (d, *J* = 5.9 Hz, 1H), 2.18 – 2.15 (m, 1H), 2.06 (s, 1H), 2.04 (d, *J* = 3.3 Hz, 1H), 2.01 (s, 1H), 1.97 (s, 1H), 1.73 (s, 1H), 1.69 (s, 1H), 1.57 (s, 3H), 1.42 (s, 1H), 1.39 (s, 1H), 1.37 (d, *J* = 4.2 Hz, 1H), 1.34 (s, 1H), 1.25 (s, 1H), 1.23 (s, 1H), 1.01 (s, 1H), 0.99 (s, 1H), 0.91 (s, 5H), 0.84 (s, 1H), 0.82 (d, *J* = 3.2 Hz, 7H). ^{13}C NMR (126 MHz, $CDCl_3$) δ 165.41, 135.34, 135.21, 132.46, 131.06, 130.56, 128.97, 81.89, 79.54, 69.80, 56.33, 51.75, 51.18, 44.70, 42.92, 39.86, 39.45, 37.12, 34.44, 33.32, 33.21, 28.77, 26.41, 23.52, 21.02, 20.76, 20.09, 19.78, 18.23, 17.71, 13.03.

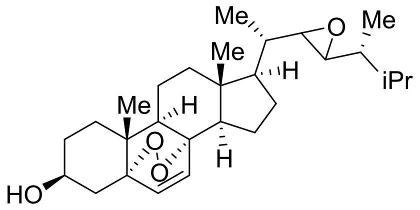

Compound 5: In a 5-mL round-bottom flask, ergosterol peroxide (**EP**, 42 mg, 0.1 mmol) was dissolved in anhydrous CH_2Cl_2 (1.0mL), which was treated with *m*CPBA (19.0 mg, 0.11 mmol) and stirred at 25°C for 16 h. The reaction was quenched with a solution of aqueous sodium thiosulfate (1.0 mL) and stirred for 30 min, then diluted with EtOAc and washed with brine (3 x 1.0 mL). The organic phase was dried over $MgSO_4$, filtered, and concentrated. The crude product was purified to afford compound **5** as a white foam (41.0 mg, 0.090 mmol, 92% yield). HRMS (ESI-TOF) calculated for $C_{28}H_{45}O_4$ ($[M+ H]^+$): 445.3318, found: 445.3313. ^1H NMR (500 MHz, $CDCl_3$) δ 6.49 (dd, *J* = 8.4, 2.8 Hz, 1H), 6.25 (dd, *J* = 8.5, 6.0 Hz, 1H), 3.96 (tt, *J* = 11.1, 5.0 Hz, 1H), 2.66 (dd, *J* = 8.4, 2.3 Hz, 1H), 2.45 (dd, *J* = 7.9, 2.3 Hz, 1H), 2.37 (dd, *J* = 8.2, 2.2 Hz, 1H), 2.14 – 2.08 (m, 1H), 1.94 (dd, *J* = 14.5, 11.4 Hz, 4H), 1.87 – 1.81 (m, 1H), 1.76 (ddd, *J* = 13.2, 6.5, 4.6 Hz, 1H), 1.66 (s, 5H), 1.55 (s, 1H), 1.51 (s, 1H), 1.49 (s, 1H), 1.39 (ddd, *J* = 19.0, 9.5, 3.3 Hz, 2H), 1.25 (s, 3H), 1.20 – 1.14 (m, 1H), 1.08 (s, 3H), 0.99 (s, 1H), 0.97 (s, 1H), 0.96 (s, 1H), 0.95 (s, 1H), 0.93 (s, 1H), 0.92 (s, 1H), 0.91 (s, 1H), 0.90 (s, 1H), 0.88 (s, 3H), 0.78 (s, 3H). ^{13}C NMR (126 MHz, $CDCl_3$) δ 135.50, 130.35, 82.11, 79.20, 66.28, 63.79, 62.77, 60.24, 56.13, 53.76, 44.83, 42.31, 42.12, 39.05, 38.08, 36.81, 36.75, 34.55, 30.86, 29.95, 27.87, 26.93, 20.32, 18.05, 15.90, 13.55, 12.60.

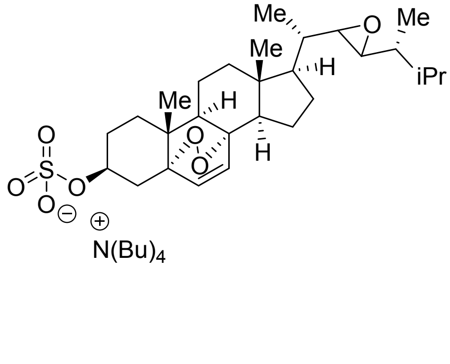

Compound 5a: To a solution of compound **5** (44.0 mg, 0.10 mmol) in dry THF (1.0 mL) was added SO_3.pyr (16.0 mg, 0.1 mmol) and the reaction mixture was stirred at 45°C for 5 h, followed by addition of tetrabutylammonium iodide (37.0 mg, 0.10 mmol) and continue stirred at this temperature for

20 mins. The above reaction mixture was then diluted with brine (1.0 mL) and saturated aqueous sodium thiosulfate (1.0 mL), followed by extraction with EtOAc (3 x 1.0 mL). The combined organic phase was dried over $MgSO_4$ and concentrated under reduced pressure to provide compound **5** (71.3 mg, 0.093 mol, 93% yield) as clear oil. HRMS (ESI-TOF) calculated for $C_{44}H_{80}NO_7S$ ([M + H]+): 766.5655, found: 766.5651. ^{1}H NMR (500 MHz, $CDCl_3$) δ 6.47 (dd, *J* = 8.5, 2.8 Hz, 1H), 6.23 (dd, *J* = 8.5, 6.2 Hz, 1H), 4.01 – 3.89 (m, 1H), 3.55 – 3.38 (m, 1H), 3.33 – 3.24 (m, 4H), 2.65 (dd, *J* = 8.4, 2.2 Hz, 1H), 2.57 (dd, *J* = 7.1, 2.3 Hz, 1H), 2.44 (dd, *J* = 7.9, 2.4 Hz, 1H), 2.41 (d, *J* = 4.4 Hz, 1H), 2.38 – 2.35 (m, 1H), 2.09 (ddd, *J* = 13.6, 5.3, 2.5 Hz, 1H), 2.03 (s, 1H), 1.99 (dt, *J* = 13.2, 4.0 Hz, 1H), 1.95 (d, *J* = 3.4 Hz, 1H), 1.94 – 1.92 (m, 1H), 1.91 (t, *J* = 2.4 Hz, 1H), 1.83 (dddd, *J* = 14.2, 6.8, 3.4, 1.6 Hz, 1H), 1.75 (ddd, *J* = 13.5, 6.8, 4.8 Hz, 1H), 1.66 (dt, *J* = 10.7, 6.8 Hz, 7H), 1.54 (s, 3H), 1.50 – 1.42 (m, 7H), 1.41 (s, 1H), 1.39 – 1.31 (m, 2H), 1.25 (s, 2H), 1.23 (dd, *J* = 4.6, 2.5 Hz, 1H), 1.20 (s, 1H), 1.19 – 1.08 (m, 2H), 1.07 (s, 1H), 1.06 (s, 1H), 1.04 – 1.02 (m, 1H), 1.01 (s, 1H), 0.99 (s, 2H), 0.98 (s, 2H), 0.96 (d, *J* = 1.4 Hz, 2H), 0.95 (s, 1H), 0.93 (d, *J* = 2.0 Hz, 2H), 0.92 (s, 1H), 0.91 (s, 1H), 0.91 (s, 1H), 0.90 (d, *J* = 1.6 Hz, 2H), 0.89 (s, 1H), 0.89 (s, 1H), 0.86 (d, *J* = 1.7 Hz, 4H), 0.84 – 0.78 (m, 1H), 0.77 (s, 3H), 0.70 – 0.59 (m, 1H). ^{13}C NMR (126 MHz, $CDCl_3$) δ 135.74, 130.53, 82.35, 79.41, 66.45, 64.01, 63.00, 60.48, 59.12, 56.34, 53.96, 51.36, 51.14, 45.06, 45.04, 42.52, 42.33, 39.39, 39.33, 39.25, 38.31, 37.02, 34.77, 31.19, 31.07, 30.40, 30.16, 30.14, 28.08, 27.15, 24.26, 21.04, 20.92, 20.53, 20.33, 19.86, 19.59, 18.64, 18.27, 16.99, 16.12, 13.84, 12.82, 12.66.

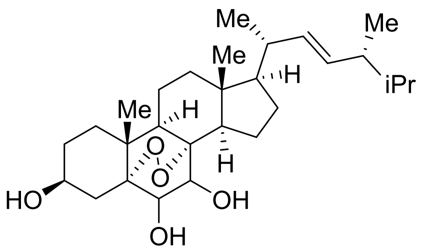

Compound 6: To a solution of ergosterol peroxide (**EP**, 42.0 mg, 0.1 mmol) in acetone/H_2O (1:1, v/v, 1.0 mL) was added OsO_4 (40.0 μL, 2.5%, wt.% in 2-methyl-2-propanol) and NMO (23.0 mg, 0.20 mmol) at 25°C, the reaction mixture was vigorously stirred at this temperature for additional 12 h, the reaction progress was monitored by TLC until completion. The above reaction mixture was then diluted with brine (1.0 mL) and extracted with EtOAc (3 x 1.0 mL). The combined organic phase was dried over $MgSO_4$ and concentrated under reduced pressure. The crude product was subjected to silica gel column chromatography (Hexane:EtOAc, 7:3 to 1:9, v/v) to provide diol compound **6** (40.0 mg, 0.087 mol, 87% yield) as clear oil. HRMS (ESI-TOF) calculated for $C_{28}H_{47}O_5$ ([M + H]$^+$): 463.3423, found: 463.3420. ^{1}H NMR (400 MHz, $CDCl_3$) δ 6.50 (d, *J* = 8.5 Hz, 1H), 6.25 (d, *J* = 8.5 Hz, 1H), 3.97 (tt, *J* = 11.0, 5.1 Hz, 1H), 3.71 (d, *J* = 4.1 Hz, 1H), 3.60 (s, 1H), 2.17 (s, 1H), 2.04 (s, 1H), 1.94 (s, 1H), 1.91 (d, *J* = 2.3 Hz, 1H), 1.82 (s, 1H), 1.71 (d, *J* = 3.7 Hz, 2H), 1.67 (s, 1H), 1.62 (s, 4H), 1.51 (s, 1H), 1.49 (s, 1H), 1.29 (s, 1H), 1.25 (s, 3H), 1.23 (s, 1H), 1.01 (s, 1H), 0.99 (s, 1H), 0.97 (s, 2H), 0.95 (s, 2H), 0.93 (s, 1H), 0.91 (s, 2H), 0.88 (d, *J* = 3.1 Hz, 6H), 0.86 (d, *J* = 3.3 Hz, 3H), 0.83 (s, 2H), 0.82 (s, 1H). ^{13}C NMR (126 MHz, $CDCl_3$) δ 135.76, 130.64, 82.36, 79.47, 73.23, 70.31, 66.56, 53.17, 51.46, 51.22, 45.31, 44.07, 41.60, 39.55, 37.08, 37.04, 34.83, 30.24, 29.97, 28.16, 23.52, 21.58, 20.98, 19.01, 18.29, 13.99, 12.63, 10.06.

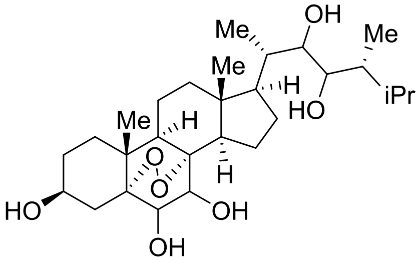

Compound 8: To a solution of ergosterol peroxide (**EP**, 42.0 mg, 0.10 mmol) in acetone/H_2O (1:1, v/v, 1.0 mL) was added OsO_4 (40.0 µL, 2.5%, wt.% in 2-methyl-2-propanol) and NMO (60.0 mg, 0.50 mmol) at 25°C, the reaction mixture was vigorously stirred at this temperature for additional 24 h, the reaction progress was monitored by TLC until completion. The above reaction mixture was then diluted with brine (1.0 mL) and extracted with EtOAc (5 x 1.0 mL). The combined organic phase was dried over $MgSO_4$ and concentrated under reduced pressure. The crude product was subjected to silica gel column chromatography (Hexane:EtOAc, 7:3 to 1:9, v/v) to provide compound **8** (38.0 mg, 0.082 mol, 82% yield) as clear oil. HRMS (ESI-TOF) calculated for $C_{28}H_{49}O_7$ ([M+ H]$^+$): 497.3478, found: 497.3481. ^{1}H NMR (500 MHz, $CDCl_3$) δ 4.24 – 4.15 (m, 1H), 3.79 – 3.67 (m, 3H), 3.60 (s, 1H), 3.34 (d, *J* = 8.1 Hz, 1H), 2.80 (d, *J* = 11.2 Hz, 1H), 2.28 (d, *J* = 6.0 Hz, 1H), 2.22 (ddd, *J* = 14.1, 4.9, 2.0 Hz, 1H), 2.12 – 2.06 (m, 1H), 1.81 – 1.78 (m, 2H), 1.71 – 1.65 (m, 3H), 1.63 – 1.48 (m, 9H), 1.39 (ddd, *J* = 14.5, 10.8, 4.3 Hz, 2H), 1.31 – 1.23 (m, 5H), 1.20 (t, *J* = 10.1 Hz, 1H), 1.02 – 0.83 (m, 18H). ^{13}C NMR (126 MHz, $CDCl_3$) δ 171.61, 83.57, 73.50, 72.89, 70.51, 69.27, 67.36, 66.84, 60.81, 55.93, 53.48, 52.33, 45.09, 40.26, 36.86, 34.28, 29.64, 27.41, 21.81, 21.43, 19.30, 18.06, 17.74, 14.57, 14.26, 12.92, 11.23, 10.31.

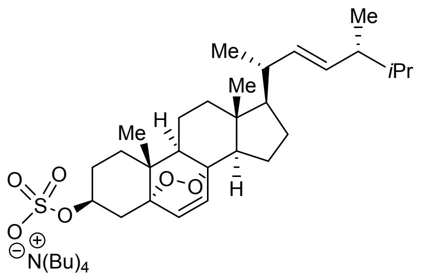

Compound 9: To a solution of ergosterol peroxide (**EP**, 42.0 mg, 0.10 mmol) in dry THF (1.0mL) was added SO_3.pyr (16.0 mg, 0.1 mmol) and the reaction mixture was stirred at 45°C for 5 h, followed by addition of tetrabutylammonium iodide (37.0 mg, 0.10 mmol) and continue stirred at this temperature for 20 min. The above reaction mixture was then diluted with brine (1.0 mL) and saturated aqueous sodium thiosulfate (1.0 mL), followed by extraction with EtOAc (3 x 1.0 mL). The combined organic phase was dried over $MgSO_4$ and concentrated under reduced pressure to provide compound **9** (65.0 mg, 0.087 mol, 87% yield) as clear oil. HRMS (ESI-TOF) calculated for $C_{44}H_{80}NO_6S$ ([M+ H]+): 750.5706, found: 750.5710. ^{1}H NMR (500 MHz, $CDCl_3$) δ 6.49 (d, J = 8.5 Hz, 1H), 6.23 (d, J = 8.5 Hz, 1H), 5.24 – 5.10 (m, 3H), 3.95 (ddd, J = 11.5, 6.5, 5.1 Hz, 1H), 3.35 – 3.29 (m, 3H), 2.13 – 1.80 (m, 12H), 1.78 – 1.64 (m, 7H), 1.62 – 1.31 (m, 17H), 1.27 – 1.19 (m, 6H), 1.03 – 0.98 (m, 8H), 0.92 – 0.86 (m, 8H), 0.84 – 0.79 (m, 11H), -0.01 (s, 1H). ^{13}CNMR (126 MHz, $CDCl_3$) δ 135.35, 135.30, 135.07, 134.98, 132.29, 132.17, 130.59, 82.04, 79.28, 66.30, 59.01, 56.06, 51.55, 50.96, 44.42, 42.64, 39.59, 39.21, 36.83, 36.79, 34.57, 32.93, 30.19, 29.98, 28.51, 24.10, 23.26, 20.74, 20.50, 19.81, 19.65, 19.50, 18.04, 17.42, 13.59, 12.74.

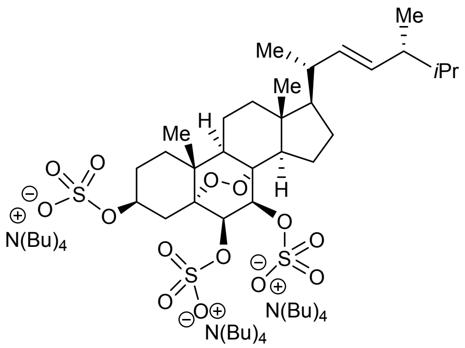

Compound 10: To a solution of ergosterol peroxide (**EP**, 42.0 mg, 0.10 mmol) in dry THF (1.0 mL) was added

SO$_3$.pyr (48.0 mg, 0.3 mmol) and the reaction mixture was stirred at 45°C for 5 h, followed by addition of tetrabutylammonium iodide (110.0 mg, 0.30 mmol) and continue stirred at this temperature for 20 mins. The above reaction mixture was then diluted with brine (1.0 mL) and saturated aqueous sodium thiosulfate (1.0 mL), followed by extraction with EtOAc (3 x 1.0 mL). The combined organic phase was dried over $MgSO_4$ and concentrated under reduced pressure to provide compound **10** (118.0 mg, 0.083mol, 83% yield) as white solid. HRMS (ESI-TOF) calculated for $C_{76}H_{152}N_3O_{14}S_3$ ([M+ H]$^+$): 1427.0436, found: 1427.0431. ^{1}H NMR (500 MHz, $CDCl_3$) δ 6.48 (dd, *J* = 8.5, 2.7 Hz, 1H), 6.23 (d, *J* = 8.5 Hz, 1H), 3.94 (dq, *J* = 11.0, 5.6 Hz, 1H), 3.71 – 3.67 (m, 1H), 3.59 (t, *J* = 3.0 Hz, 1H), 3.41 – 3.28 (m, 19H), 2.25 (s, 1H), 2.12 – 1.76 (m, 12H), 1.68 (dq, *J* = 12.2, 7.6 Hz, 22H), 1.62 – 1.50 (m, 5H), 1.49 – 1.40 (m, 25H), 1.24 (d, *J* = 1.3 Hz, 10H), 1.16 – 1.03 (m, 2H), 1.00 (t, *J* = 7.4 Hz, 29H), 0.95 (dd, *J* = 6.8, 3.7 Hz, 3H), 0.91 – 0.78 (m, 16H), -0.02 (s, 2H). ^{13}C NMR (126 MHz, $CDCl_3$) δ 135.98, 135.95, 130.91, 130.84, 125.86, 82.59, 82.57, 79.68, 73.37, 70.46, 66.73, 59.57, 53.37, 53.36, 51.67, 51.44, 45.52, 44.29, 41.86, 41.80, 39.76, 37.29, 37.25, 35.06, 30.67, 30.48, 30.16, 28.37, 27.38, 24.65, 23.73, 21.82, 21.20, 20.14, 19.22, 18.52, 18.50, 14.23, 14.09, 12.84, 12.75, 11.20, 10.29, 0.34.

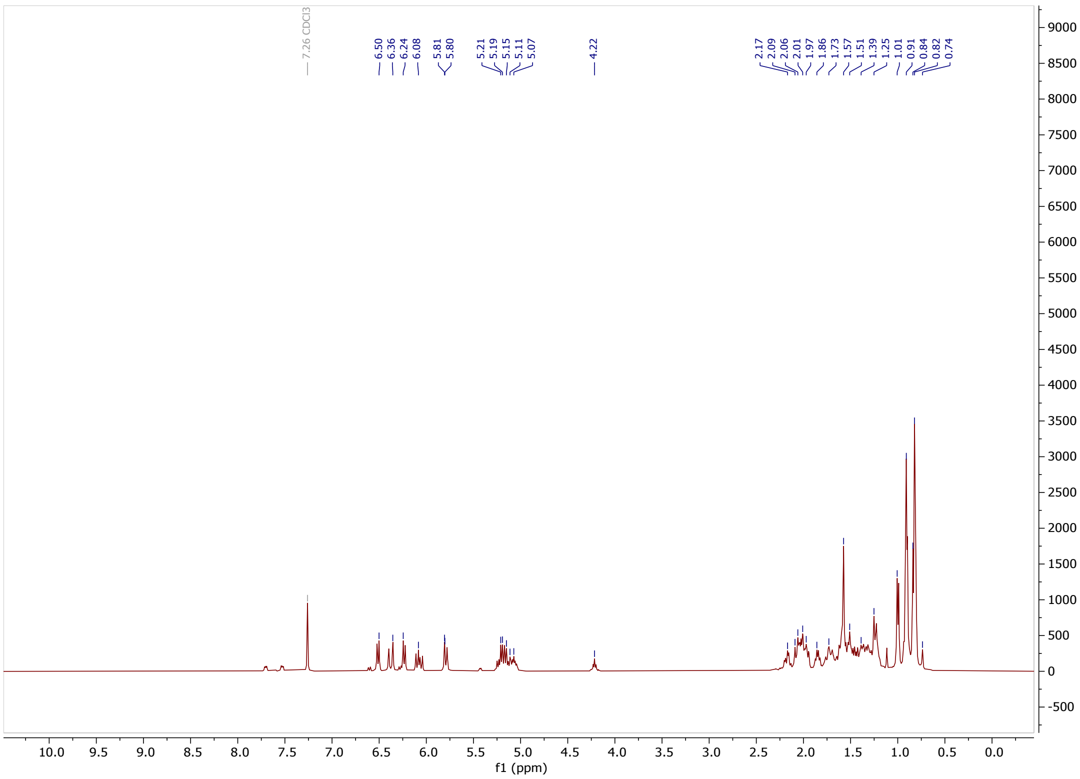

Figure S1. ^{1}H NMR Spectrum of **Compound 4**.

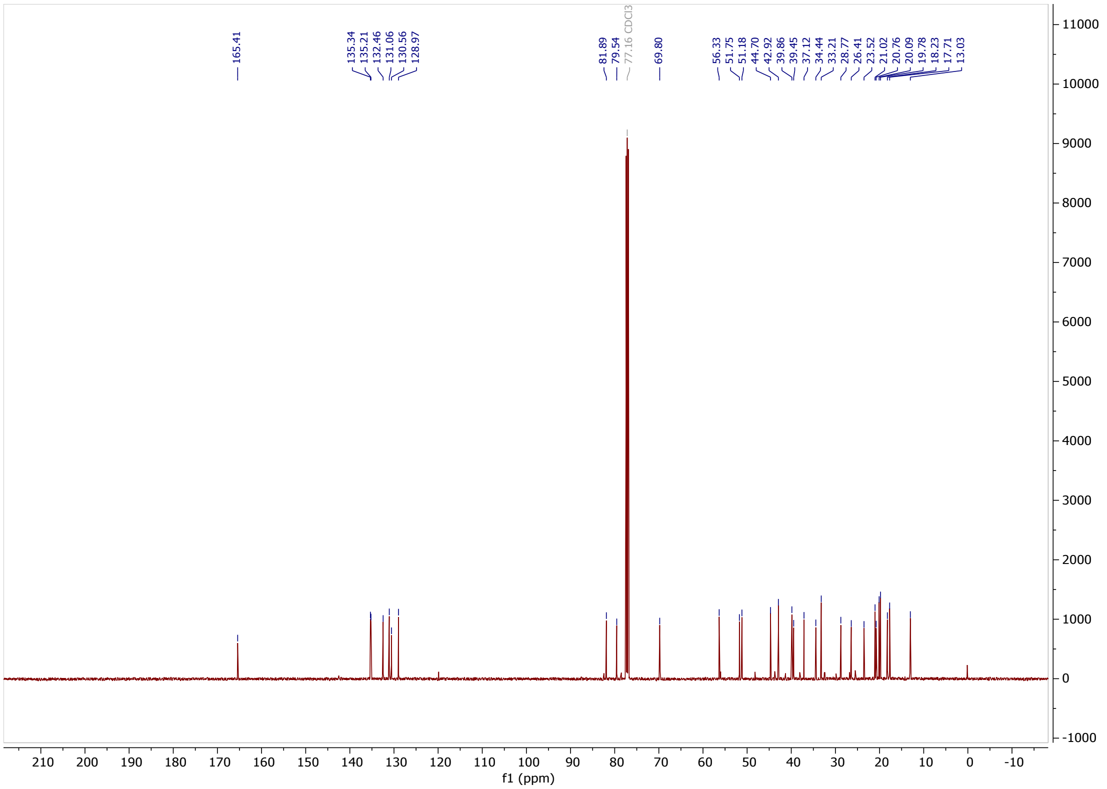

Figure S2. ^{13}C NMR Spectrum of **Compound 4**.

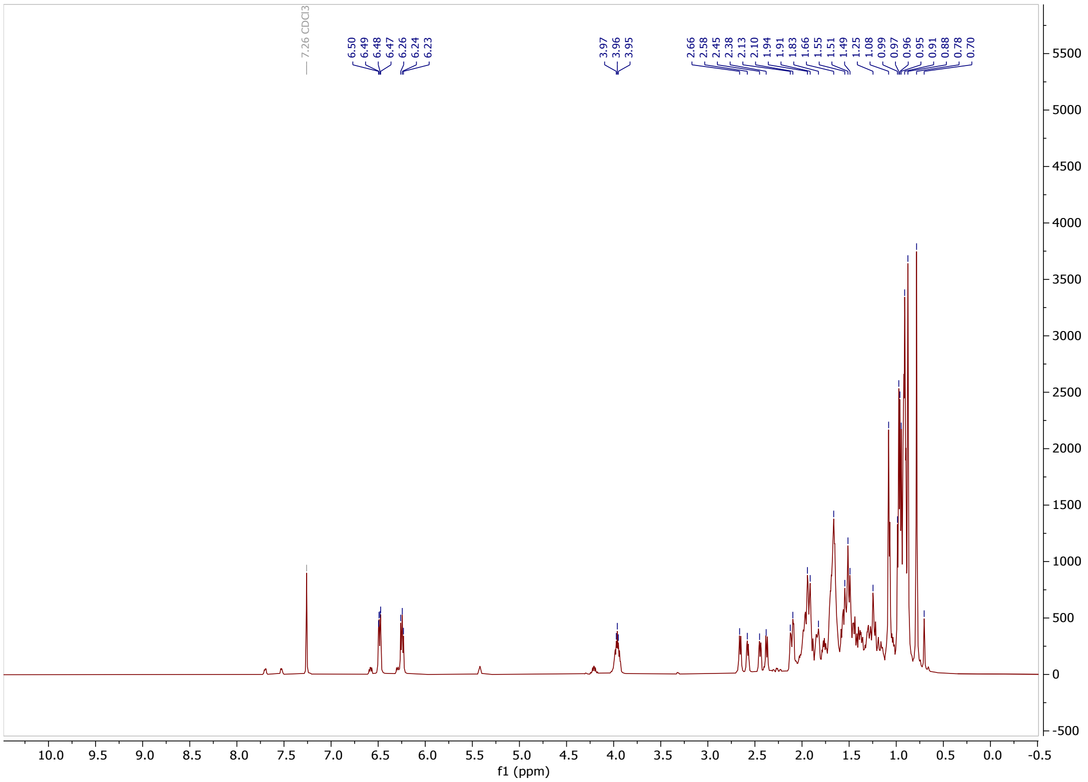

Figure S3. ^{1}H NMR Spectrum of **Compound 5**.

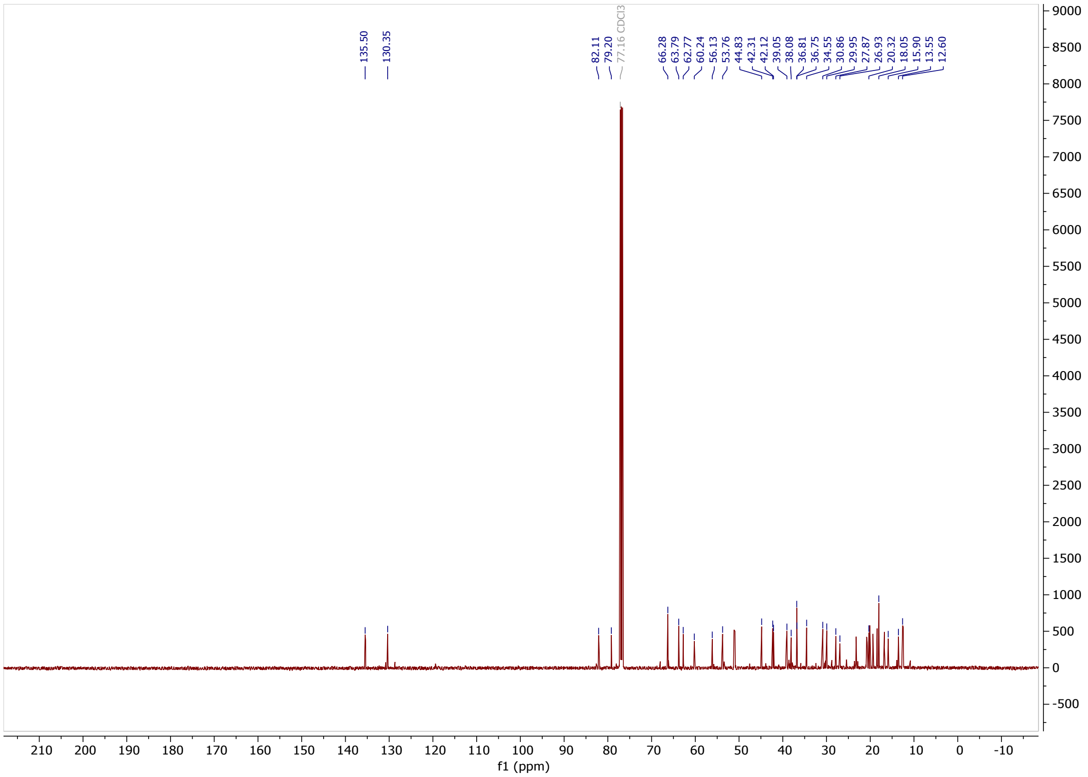

Figure S4. ^{13}C NMR Spectrum of **Compound 5**.

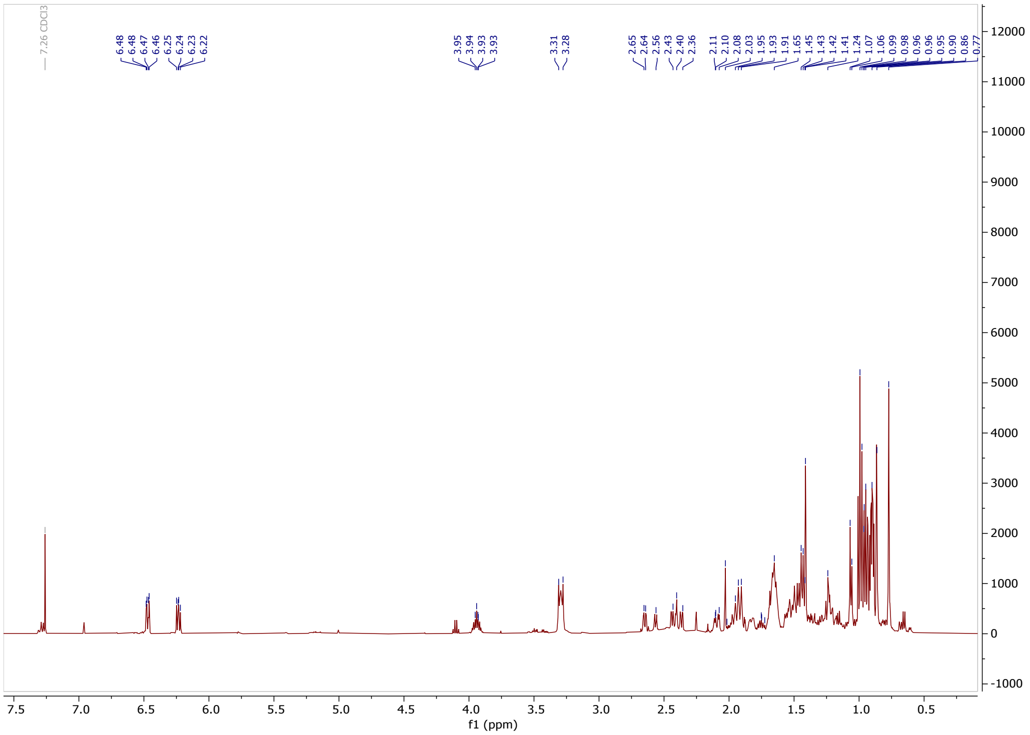

Figure S5. ^{1}H NMR Spectrum of **Compound 5a**.

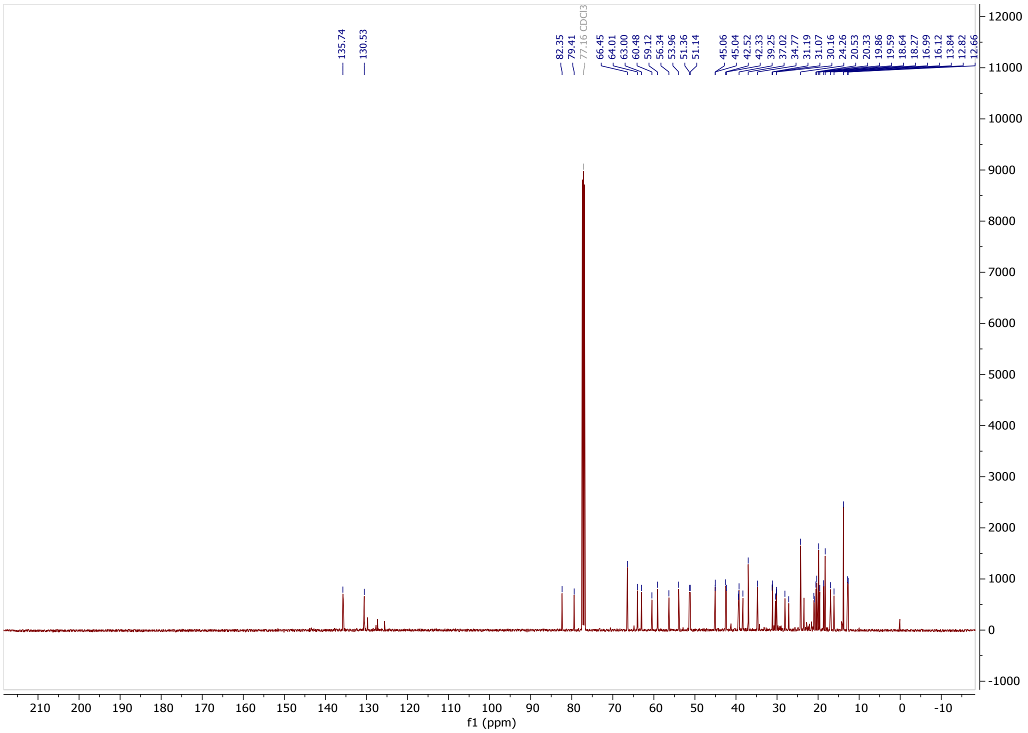

Figure S6. ^{13}C NMR Spectrum of **Compound 5a**.

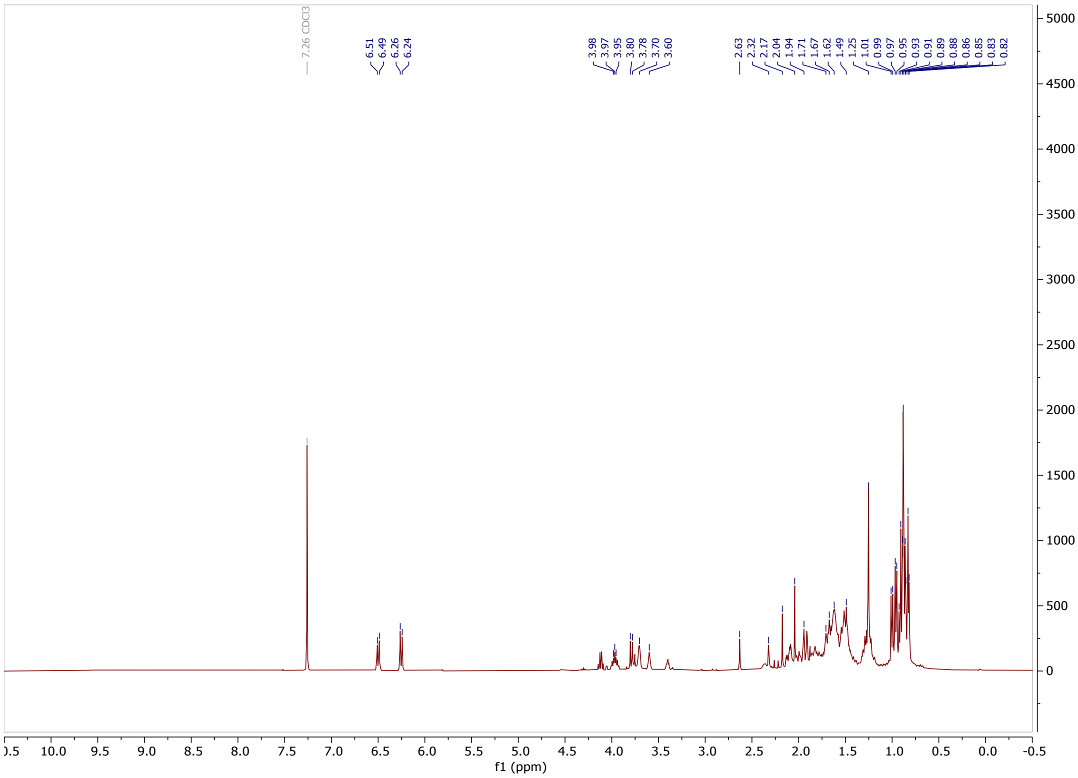

Figure S7. ^{1}H NMR Spectrum of **Compound 6**.

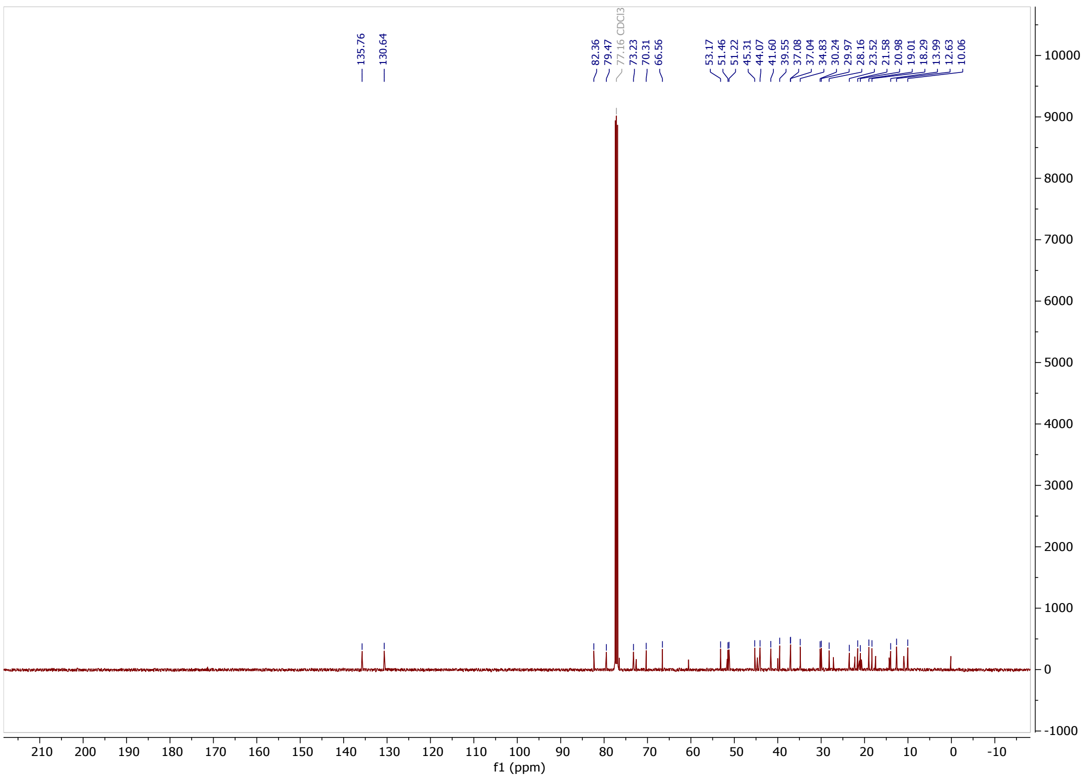

Figure S8. ^{13}C NMR Spectrum of **Compound 6**.

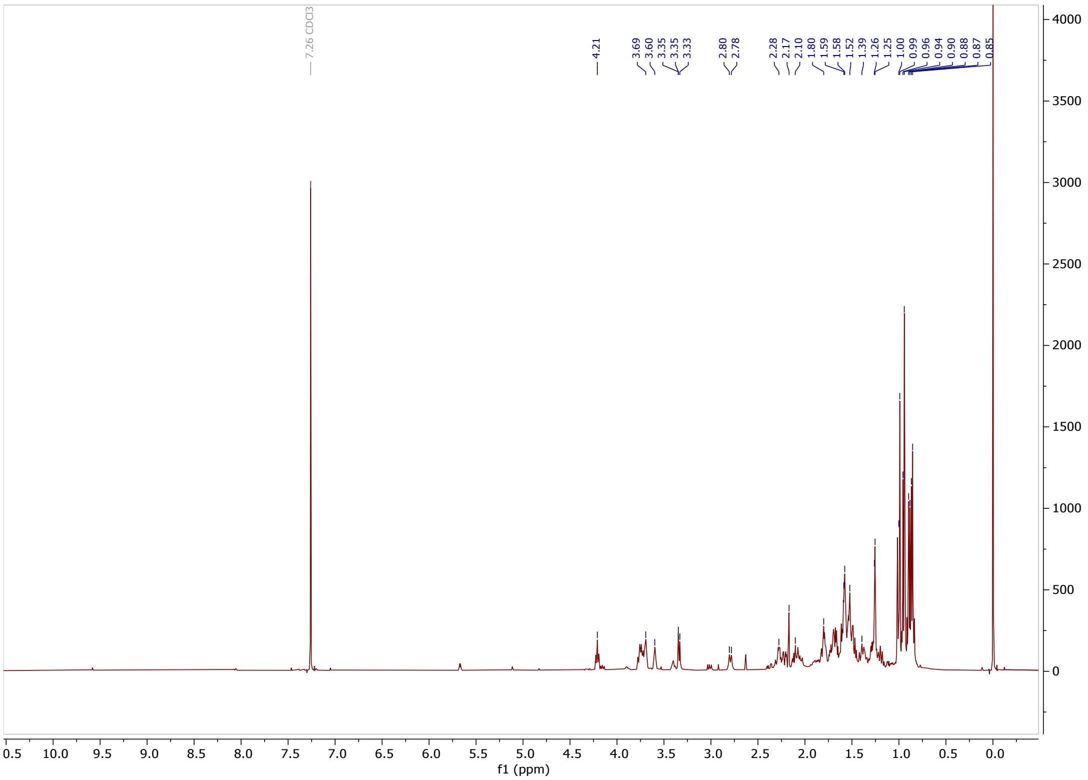

Figure S9. ^{1}H NMR Spectrum of **Compound 8**.

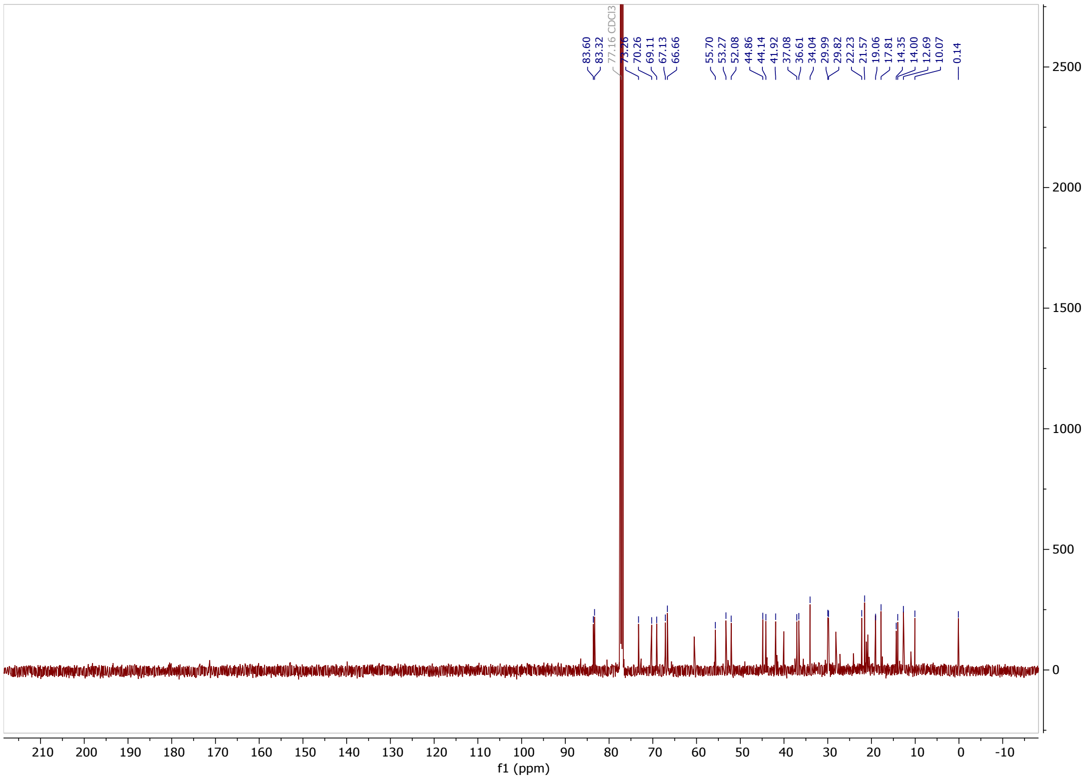

Figure S10. ^{13}C NMR Spectrum of **Compound 8**.

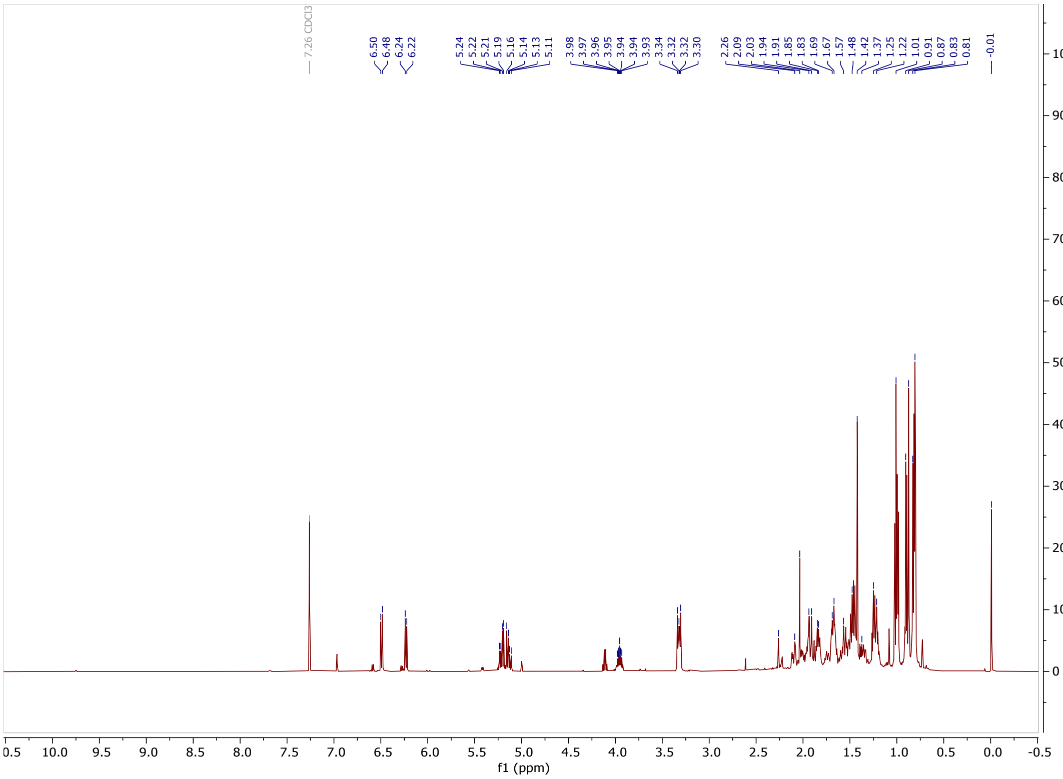

Figure S11. ^{1}H NMR Spectrum of **Compound 9**.

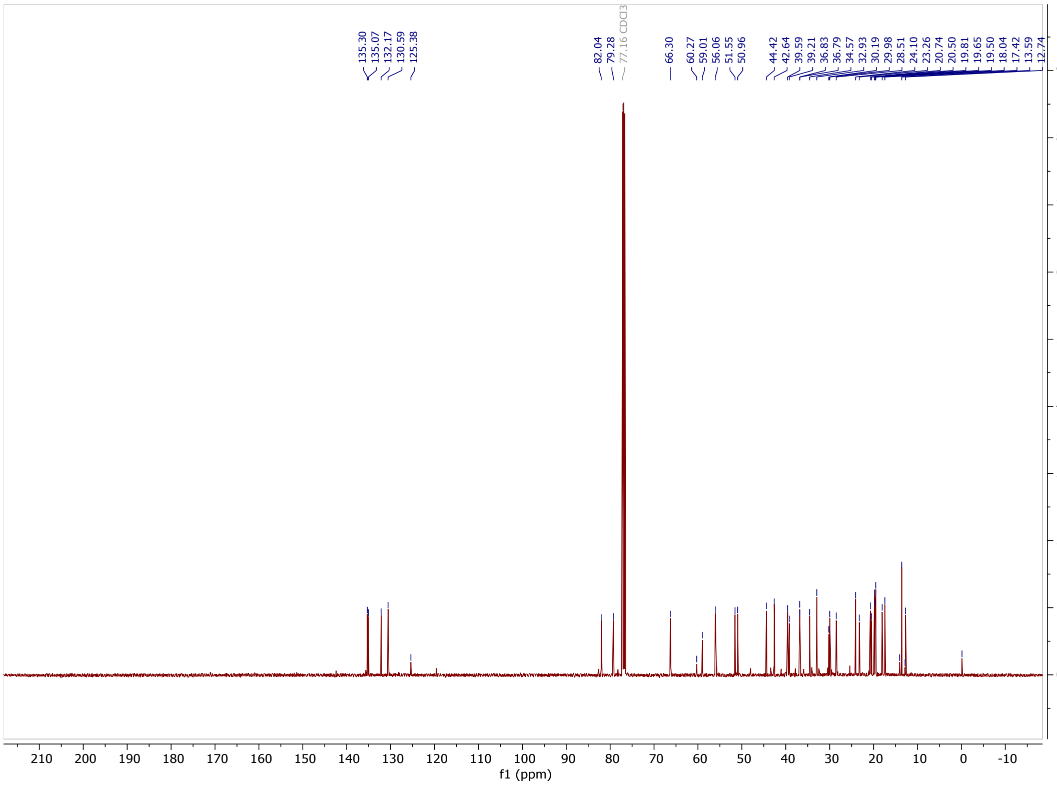

Figure S12. ^{13}C NMR Spectrum of **Compound 9**.

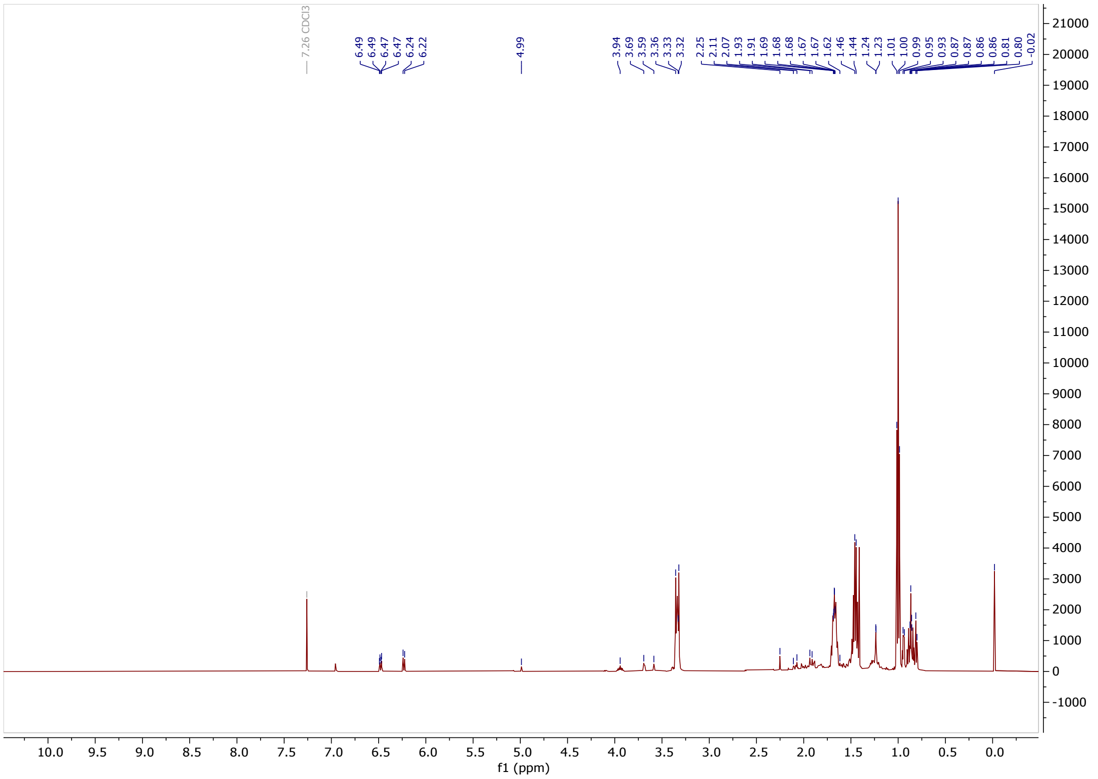

Figure S13. ^{1}H NMR Spectrum of **Compound 10**.

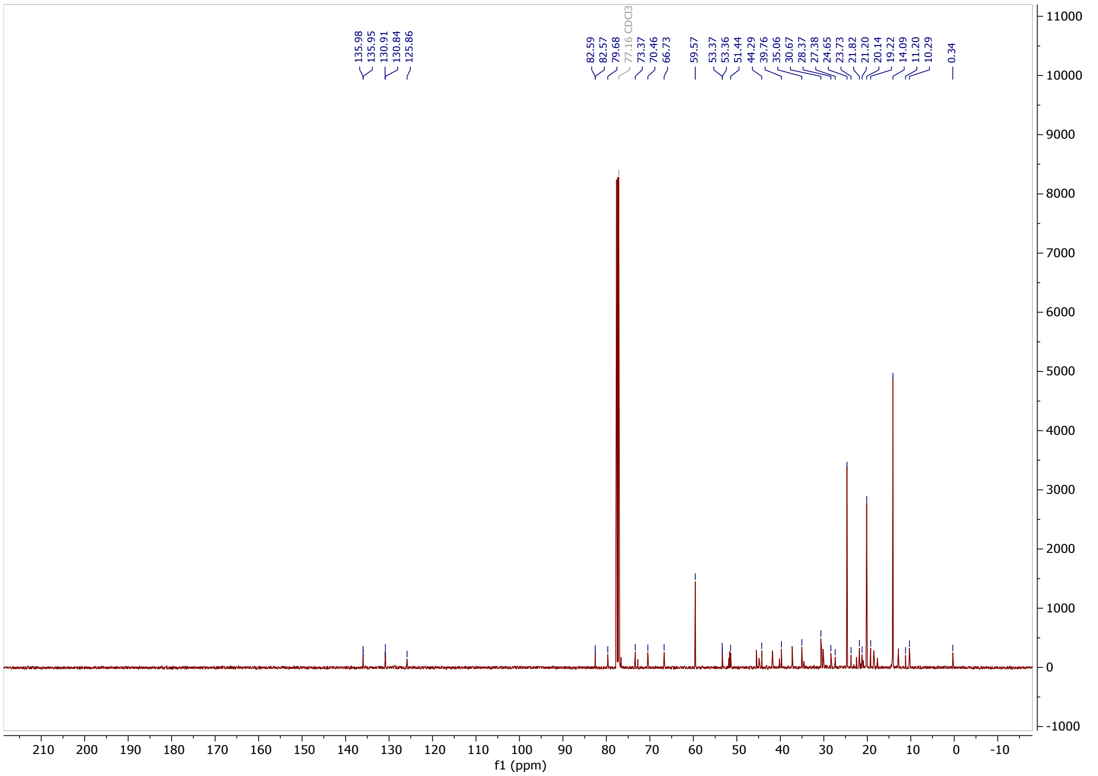

Figure S14. ^{13}C NMR Spectrum of **Compound 10**.
